# Baseline analysis of *Mycoplasma mycoides* subsp. *mycoides* antigens as targets for a DIVA assay for use with a subunit vaccine for contagious bovine pleuropneumonia

**DOI:** 10.1186/s12917-020-02453-w

**Published:** 2020-07-10

**Authors:** Harrison O. Lutta, David Odongo, Arshad Mather, Jose Perez-Casal, Andrew Potter, Volker Gerdts, Emil M. Berberov, Tracy Prysliak, Martina Kyallo, Alexander Kipronoh, Moses Olum, Roger Pelle, Jan Naessens

**Affiliations:** 1 Kenya Agricultural and Livestock Research Organization, Biotechnology Research Institute, P.O. Box 14733-00800, Nairobi, Kenya; 2 School of Biological Sciences, University of Nairobi, P.O. Box 30197-00100, Nairobi, Kenya; 3 Agricultural Research Council - Onderstepoort Veterinary Research, Private Bag X5, Onderstepoort, Pretoria, 0110 South Africa; 4 Vaccine and Infectious Disease Organization-International Vaccine Centre (VIDO-InterVac), University of Saskatchewan, 120 Veterinary Rd, Saskatoon, SK S7N 5E3 Canada; 5 Biosciences eastern and central Africa-International Livestock Research Institute, Old Naivasha Road, P.O. Box 30709-00100, Nairobi, Kenya; 6 Kenya Agricultural and Livestock Research Organization, Veterinary Science Research Institute, P.O. Box 32-00902, Kikuyu, Kenya

**Keywords:** Antigens, Cattle, CBPP, DIVA, Mycoplasma mycoides

## Abstract

**Background:**

*Mycoplasma mycoides* subsp. *mycoides* (*Mmm*) is the causative agent of contagious bovine pleuropneumonia in cattle. A prototype subunit vaccine is being developed, however, there is currently no diagnostic test that can differentiate between infected cattle and those vaccinated with the prototype subunit vaccine. This study characterized *Mmm* proteins to identify potential antigens for use in differentiating infected from vaccinated animals.

**Results:**

Ten *Mmm* antigens expressed as recombinant proteins were tested in an indirect ELISA using experimental sera from control groups, infected, and vaccinated animals. Data were imported into R software for analysis and drawing of the box and scatter plots while Cohen’s Kappa assessed the level of agreement between the *Mmm* antigens. Two vaccine antigens (MSC_0499 and MSC_0776) were superior in detecting antibodies in sera of animals vaccinated with the subunit vaccines while two non-vaccine antigens (MSC_0636 and LppB) detected antibodies in sera of infected animals showing all clinical stages of the disease. Sensitivity and specificity of above 87.5% were achieved when the MSC_0499 and MSC_0636 antigens were tested on sera from vaccinated and infected animals.

**Conclusions:**

The MSC_0499 and MSC_0776 antigens were the most promising for detecting vaccinated animals, while MSC_0636 and LppB were the best targets to identify infected animals. Further testing of sera from vaccinated and infected animals collected at different time intervals in the field should help establish how useful a diagnostic test based on a cocktail of these proteins would be.

## Background

*Mycoplasma mycoides* subsp. *mycoides* (*Mmm*), the causative pathogen of contagious bovine pleuropneumonia (CBPP) in cattle, belongs to the classical ‘*Mycoplasma mycoides* cluster’ [1]. Clinically, CBPP manifests as either acute, sub-acute, or chronic forms. The acute and sub-acute forms are characterized by rapid breathing, fever, nasal discharge, cough, and sudden death; whereas the chronic stage of infection is characterized by weight loss and cough on exertion. During the acute phase of the disease, autonomous lung lesions and pleural fluid are often observed on post mortem examination [2]. CBPP financial losses and control costs are estimated at €44.8 million per annum in Africa [3]. To help combat the disease, two live attenuated vaccines namely T1/44 and T1/SR, are currently in use. A complement fixation test (CFT) and a competitive ELISA (c-ELISA) are the only prescribed World Organization for Animal Health (OIE) serological diagnostic tests to work with the T1/44 and T1/SR live-attenuated vaccines. The approved live attenuated vaccines and prescribed diagnostic tests have shortcomings, which necessitates the development of more effective vaccines and diagnostics.

To develop a vaccine and a supporting diagnostic that can differentiate CBPP-infected from vaccinated cattle (DIVA), we used a reverse vaccinology approach described in Perez-Casal et al.*,* [4] to identify 28 potential *Mmm* vaccine antigens from the Gladysdale [5] and PG1 [6] genomes for a candidate subunit vaccine and a DIVA assay. Male Boran cattle in a study by Nkando et al.*,* [7] were immunized using pools of five antigens previously identified [4], followed by a challenge with the Afadé *Mmm* strain. Two of the groups immunized with five proteins each showed protection after the *Mmm* challenge [7]. Moreover, seventeen immunogenic *Mmm* proteins evaluated in a cocktail indirect ELISA (iELISA) in a study by Heller et al.*,* [8], displayed strong serological responses and high disease specificity as confirmed in earlier studies [9–13]. MSC_0136 and MSC_0636 showed sensitivity and specificity of above 85.6 and 96.4%, respectively [8]. In 2018, screening of sera from naïve and CBPP-infected cattle using CFT and iELISA revealed that the iELISA based on the LppB *Mmm* antigen was more sensitive than CFT, which is considered a gold standard in the diagnosis of CBPP [14].

The development of subunit vaccines will allow the development of CBPP diagnostic tests that can differentiate infected from vaccinated animals. Sera from cattle immunized with a live attenuated vaccine could test positive for many *Mmm* antigens. Detection of antibodies against proteins not present in the subunit vaccine would discriminate between cattle immunized with a live-attenuated vaccine or exposed to *Mmm* in the field, and those vaccinated with a subunit vaccine containing known *Mmm* antigens. This study aimed to identify diagnostic antigens of *Mmm* capable of differentiating cattle infected with CBPP from those vaccinated with the subunit vaccines for the control of CBPP. The control of CBPP would reduce morbidity and mortality in cattle and tremendously improve the revenue of pastoral communities.

## Results

### Selection of proteins for indirect ELISA

Six *Mmm* proteins were evaluated as vaccine antigens in the prototype subunit vaccines, and four non-vaccine antigens selected based on their potential to detect infected cattle, as reported in previous studies were tested against sera from control groups, CBPP-infected and subunit vaccinated animals (Table 2).

### Characterization of sera from naive and CBPP infected cattle

Sera from naïve and CBPP infected cattle were tested against the non-vaccine antigens. None of the animals seroconverted before infection with *Mmm* (Table 3). When non-vaccine antigens were used to detect antibodies in sera from animals challenged with *Mmm*, MSC_0636 and LppB detected more animals as *Mmm* positive than MSC_0397 and MSC_0653. The iELISA results showed that MSC_0636 detected 6/8, 4/8, and 7/8 animals in acute, subacute, and chronic stages of the disease, respectively, while LppB detected 4/8, 5/8, and 7/8 animals. However, MSC_0397 detected 3/8, 2/8, and 5/8 animals in acute, subacute, and the chronic stages of the disease, respectively, while MSC_0653 detected 3/8, 4/8 and 7/8 animals. When non-vaccine antigens were tested on sera from subunit-vaccinated cattle to establish whether they could discriminate these from the CBPP-infected cattle, MSC_0653 detected antibodies in sera from vaccinated cattle, rendering it unsuitable for a DIVA diagnostic test. MSC_0636 showed a significance difference (*P* < 0.05) in discriminating infected from vaccinated animals, as did LppB (Additional files 1 and 2). Thus, these two antigens (MSC_636 and LppB) appear suitable for a DIVA diagnostic test.

### Characterization of sera from control group and subunit vaccinated cattle

**Fig. 1:**
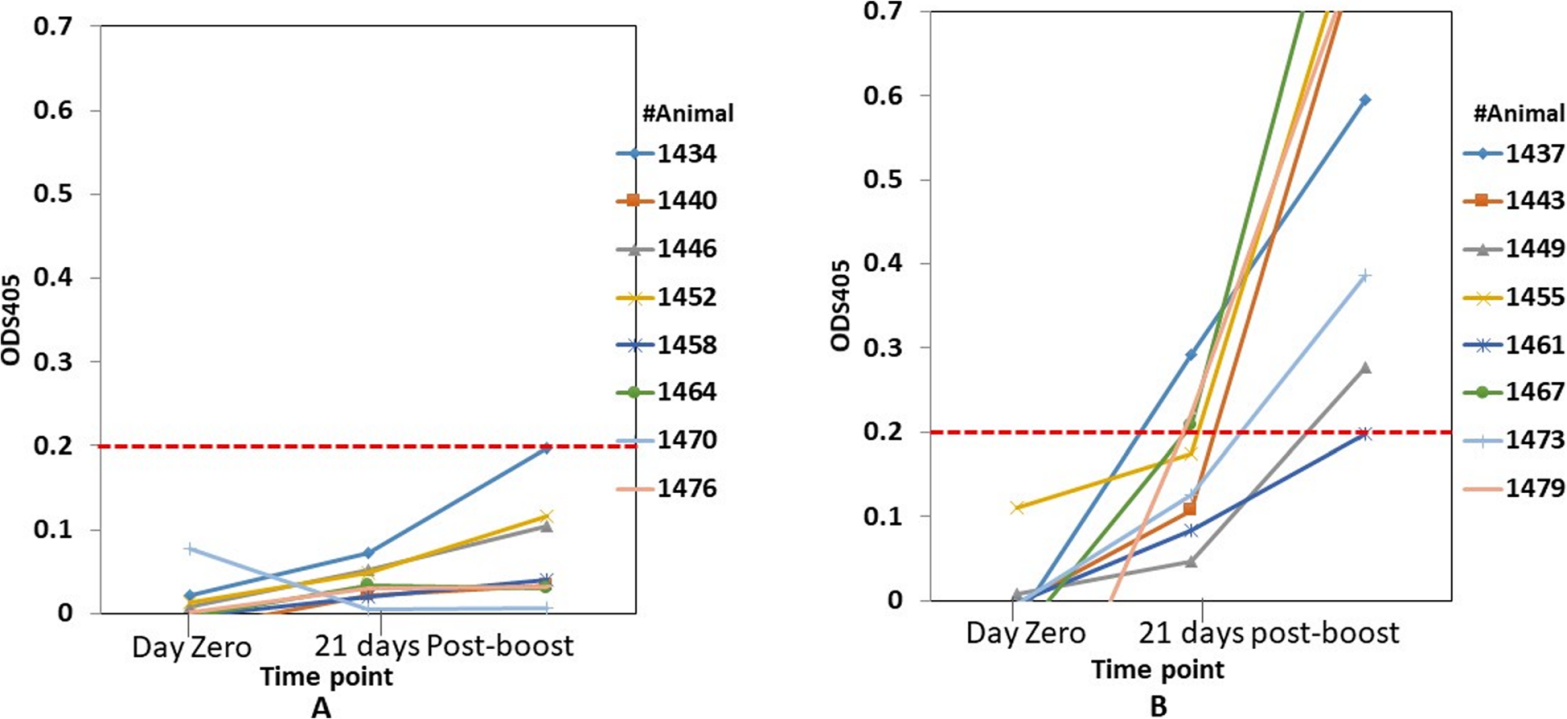
Representative iELISA results of the control group (**a**), and those formulated with test antigens (**b**). The iELISA results were based on MSC_0431 *Mmm* antigen screened on sera post-boost (time point at second immunization). Seroconversion was observed in the group formulated with the test antigens but not the control group

To define vaccine antigens that were targeted by antibody responses in naive and subunit vaccinated cattle, we determined the antibody responses against antigens in an experimental subunit vaccine. Antibodies were detected against four out of five antigens (MSC_0136, MSC_0431, MSC_0499, and MSC_0776) in the group C vaccine (Table 1) at or above the titer of 1:1600. None of the animals seroconverted before vaccination (Table 3). Figure 1 shows representative iELISA results using MSC_0431 from sera from the control group and the group vaccinated with the test antigens. The trend shown in Fig. 1 with MSC_0431 was also observed using other vaccine antigens, MSC_0136, MSC_0499, and MSC_0776 in the subunit vaccine group C (data not shown). However, when vaccine antigens were tested on sera from CBPP-infected cattle to establish whether they could discriminate these from subunit-vaccinated cattle, MSC_0431 detected antibodies in sera from infected cattle, rendering it unsuitable for a DIVA diagnostic test. The results with MSC_0499 when used to test sera from vaccinated cattle, showed a significance difference (*P* < 0.05) to those from infected animals, as did those using MSC_0776, thus making the two antigens suitable for a DIVA test (Additional files 1 and 2).

**Table 1:** Details of trial vaccines and control

*Group*	*Antigen (50* μg*/dose)*	*Adjuvant*	*No. of cattle (n)*
*A*	None (Control group)	Montanide ISA61 VG	8
*B*	MSC_0136, MSC_0431, MSC_0499 and MSC_0775	Montanide ISA61 VG	8
*C*	MSC_0136, MSC_0431, MSC_0499, MSC_0776 and MSC_0957	Montanide ISA61 VG	8

### Determination of cut-off point, diagnostic sensitivity, and specificity of *Mmm* antigens

Serum samples from naïve, CBPP-infected and vaccinated cattle returned a mean ± standard deviation OD values of 0.10 ± 0.03 (OD range of 0.07–0.19), 0.23 ± 0.14 (OD range of 0.15–0.66), and 0.24 ± 0.20 (OD range of 0.15–0.85), respectively. The change point for negative controls was as good as the cut-off point at an OD of ≤0.2 at a wavelength of 405 nm, at a titer of 1:1600 for vaccinated animals and 1:400 for infected cattle. There was a poor index value among the iELISA based on 10 *Mmm* antigens on sera from CBPP-infected, and subunit-vaccinated cattle, respectively (Cohen’s Kappa statistic k = 0.398; 0.239, Table 2). The Cochran’s Q test showed that the diagnostic sensitivity of MSC_0636, LppB, MSC_0499, and the MSC_0776 differed significantly from the rest of the antigens on sera from CBPP-infected and subunit-vaccinated cattle, respectively (Q = 41.013; df = 9, *p* < 0.001; Q = 54.419, df = 9, *p* < 0.001).

**Table 2:** Proteins used in this study

*Name*	*Description*	*Size (kDal)*	*References*
Vaccine antigens^a^			
MSC_0136	Hypothetical lipoprotein	66	11; 12
MSC_0431	Prolipoprotein	70	11; 12
MSC_0499	Prolipoprotein	111	4
MSC_0775	Prolipoprotein	81	4
MSC_0776	Prolipoprotein	120	4
MSC_0957	Prolipoprotein	79	4
Non-vaccine antigens^b^			
MSC_0397	Prolipoprotein	45	10; 11
MSC_0636	Hypothetical lipoprotein	50	12
MSC_0653	Prolipoprotein	75	11
LppB	Lipoprotein	27	24

^a^Vaccine antigens refer to proteins used to formulate prototype subunit vaccines

^b^Non-vaccine antigens refer to proteins selected based on their potential to detect CBPP-infected cattle as found in the literature

**Fig. 2:**
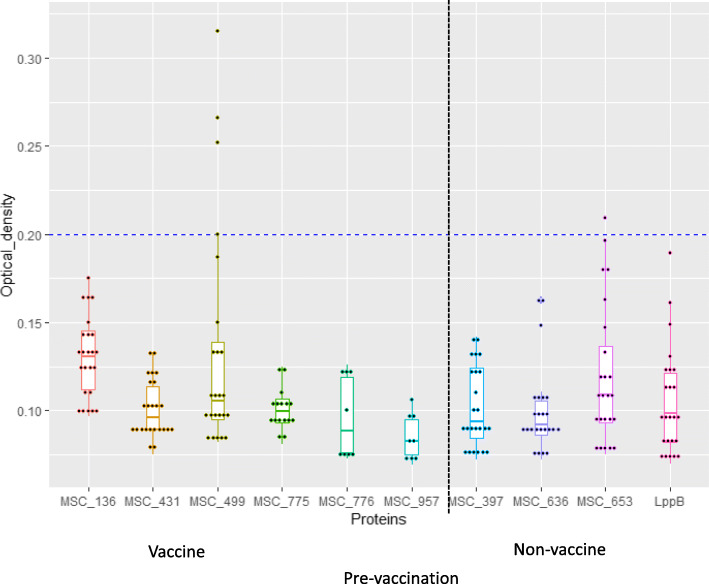
Box and scatter plots showing ODs for different *Mmm* antigens screened against sera pre-vaccination. None of the antigens showed activity with sera from naïve, pre-vaccinated animals at an OD cut-off of 0.2

**Fig. 3:**
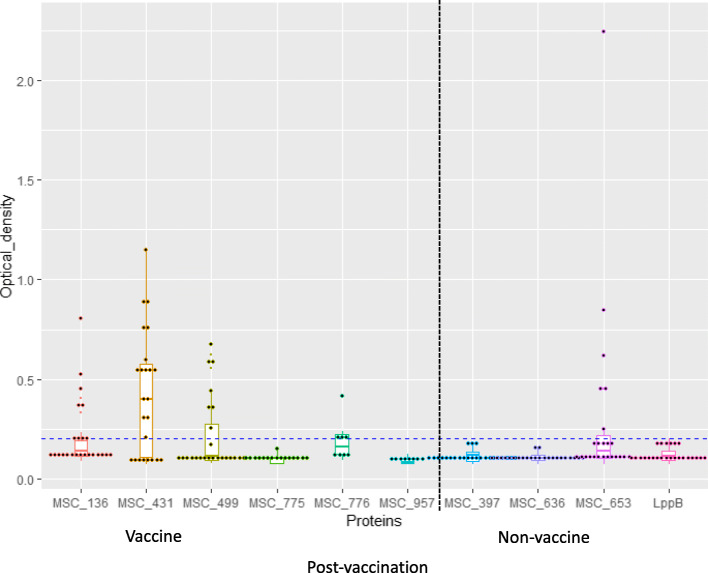
Box and scatter plots representing ODs for *Mmm* antigens screened against sera from vaccinated cattle. The horizontal blue dotted line is at OD = 0.2. Although MSC_0653 was a non-vaccine antigen and not part of the prototype vaccines, the antigen detected antibodies in sera from vaccinated animals

**Fig. 4:**
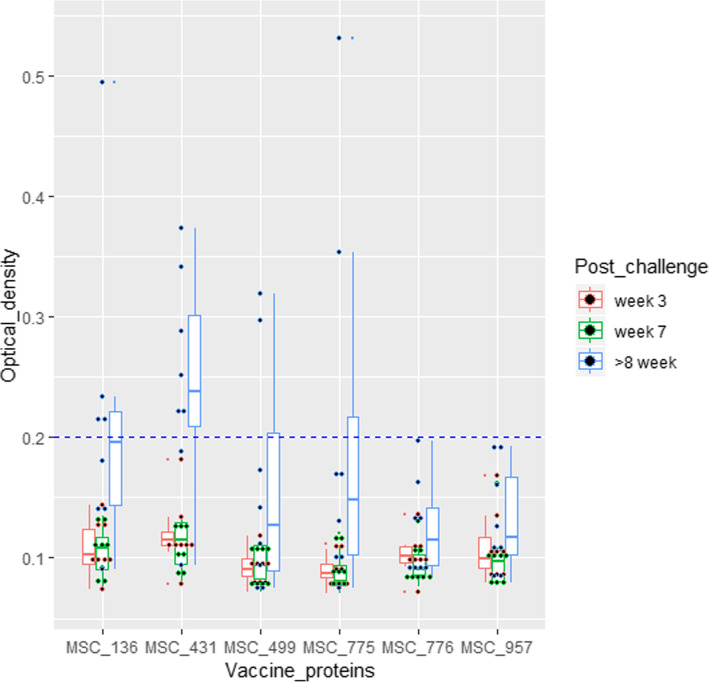
Box and scatter plots showing ODs of vaccine antigens screened against sera from CBPP-infected cattle. Although MSC_0431 and MSC_0136 were part of the prototype vaccines, the antigens detected more antibodies in sera from chronically infected animals at an OD cut-off 0.2

For the vaccine antigens, there were no significant variations in medians of ODs between *Mmm* antigens on sera from the control/naive group (Fig. 2)*.* However, there were significant variations in medians of ODs between *Mmm* antigens post-vaccination with the prototype recombinant vaccines. MSC_0499 showed a statistically significant ability to differentiate vaccinated from infected animals as did MSC_0776 (Figs. 3 and 4, Additional file 3).

**Fig. 5:**
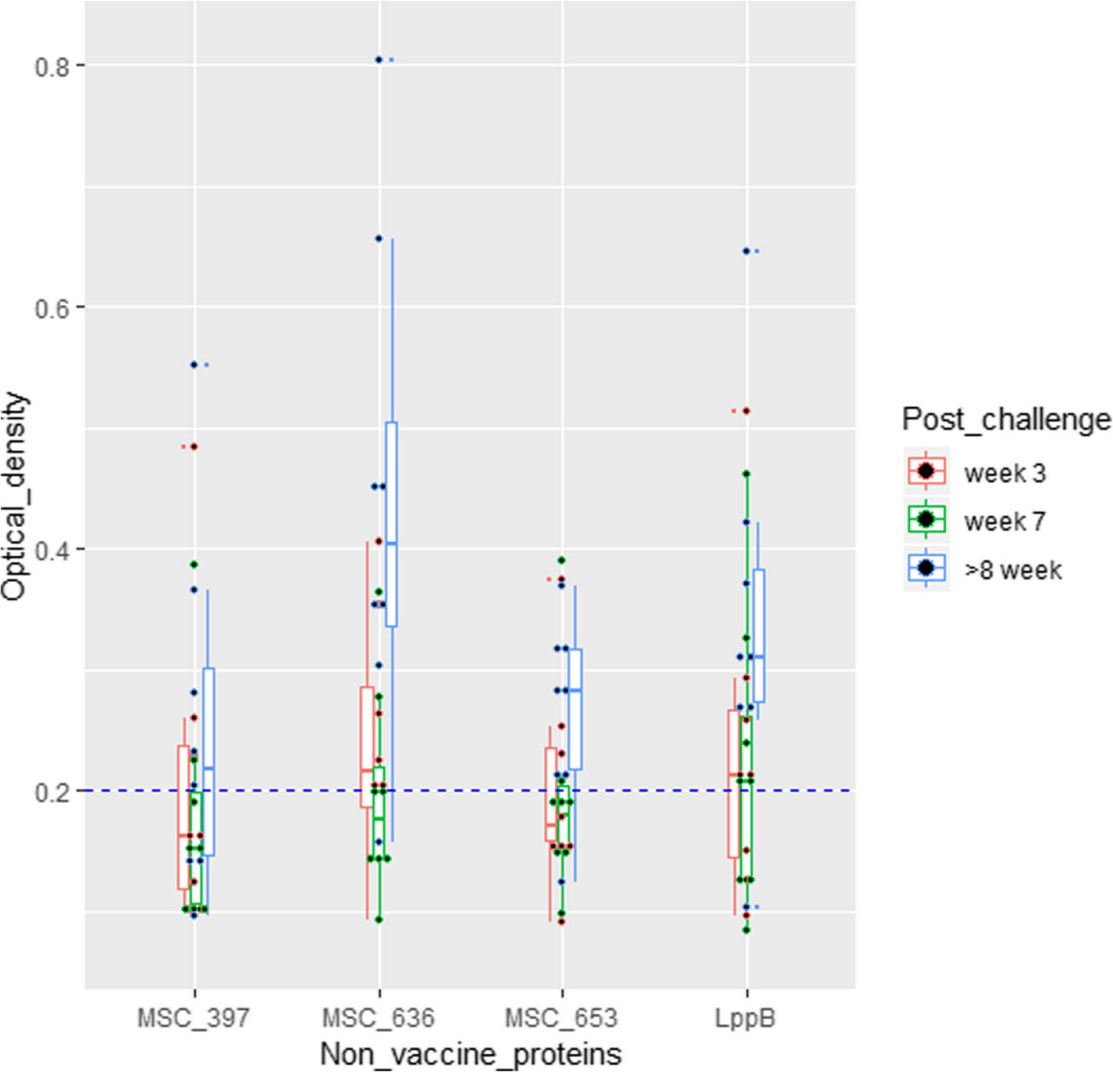
Box and scatter plots showing ODs of non-vaccine antigens screened against sera from CBPP-infected cattle. MSC_0636 significantly detected antibodies in sera from infected cattle followed by LppB. As expected, the highest seroconversion of animals was observed in the chronic disease stage (blue boxes) at an OD cut-off 0.2

When the non-vaccine antigens were assessed, MSC_0636 showed a statistically significant ability to differentiate infected from vaccinated animals, especially in the chronic clinical phase as did LppB (Figs. 3 and 5, Additional file 4). MSC_0499, MSC_0776, MSC_0636, and LppB were therefore, determined to be the best antigens for a DIVA diagnostic, as they yielded specificities of 100% and sensitivities of between 62.5–100% (Table 3, Additional files 3 and 5).

**Table 3:** ^a^ELISA results, sensitivities and specificities of antigens on sera from naive, vaccinated and CBPP-infected cattle

	Test (Antigen)	Control^b^		Sera (21 days) after boost		Se (%)	Sp (%)	PPV (%)	NPV (%)
		+Ve	-Ve	+Ve	-Ve				
Vaccine	MSC_0136	0/8	8/8	7/8	1/8	87.5	100	100	88.9
	MSC_0431	0/8	8/8	8/8	0/8	100	100	100	100
	MSC_0499	0/8	8/8	8/8	0/8	100	100	100	100
	MSC_0775	0/8	8/8	7/8	1/8	87.5	100	100	88.9
	MSC_0776	0/8	8/8	5/8	3/8	62.5	100	100	72.7
	MSC_0957	0/8	8/8	0/8	8/8	0	100	0	50
				*Sera (8–20 weeks) after infection*					
Non-vaccine	MSC_0397	0/8	8/8	5/8	3/8	62.5	100	100	72.7
	MSC_0636	0/8	8/8	7/8	1/8	87.5	100	100	88.9
	MSC_0653	0/8	8/8	7/8	1/8	87.5	100	100	88.9
	LppB	0/8	8/8	7/8	1/8	87.5	100	100	88.9

^a^This table represents results through the use of sera from naïve, CBPP-infected, and subunit-vaccinated cattle only. +Ve., positive; −Ve., negative. Cohen’s kappa index value, classified as very good (1–0.76), good (0.75–0.61), acceptable (0.6–0.4), and poor (<0.4)

^b^All CBPP-negative serum samples obtained from a group of 8 cattle (*n =* 8) were correctly identified as negative

## Discussion

Recently, our laboratories developed subunit vaccines using *Mmm* recombinant antigens and demonstrated that they elicit protective immunity against experimental endotracheal challenge with virulent *Mmm* Afade strain [7]. In Phase 1, the study tested the protective effect of 14 pools of five recombinant antigens in a CBPP challenge, and three of these pools showed clear indications of a reduction in the pathological index [7]. In Phase II, different formulations of these recombinant vaccines were evaluated by their immune responses as described by Nkando et al.*,* [7]. In this study, the *Mmm* antigens were used to analyze antibody responses in cattle infected with *Mmm* Afade strain, those vaccinated with the subunit vaccines, and naïve control groups to develop a DIVA diagnostic test.

Several studies describe the development of DIVA diagnostics/vaccines for *M. bovis* [15–17]. Zhang et al.*,* [17] used the *M. bovis*-150 strain to develop the live attenuated vaccine that elicited protection against a challenge with the virulent strain of *M. bovis* HB0801. DIVA assays using sodium thiocyanate (NaSCN) or recombinant proteins assay for use alongside the *M. bovis*-150 live attenuated vaccine have been reported [15, 16]. Han et al. [15], used NaSCN in a competitive iELISA for the detection of IgG. Khan et al.*,* [16], used proteomic techniques to identify and characterize membrane-associated proteins of *M. bovis* HB0801 and its attenuated strain (*M. bovis*-150). The results identified a putative lipoprotein encoded by a functionally unknown gene MbovP730 that demonstrated high sensitivity and specificity in an IgG iELISA. A Western blot test confirmed that MbovP730 is absent in attenuated *M. bovis*-150, indicating that this antigen could be used to develop a DIVA assay.

In a study to establish a DIVA diagnostic test to be used in combination with the CBPP live-attenuated vaccine, a very immunogenic antigen, LppQ was characterized and an LppQ-knock-out derivative of the T1/44 live attenuated vaccine strain, the T1LppQ-MT1 was created [18–20]. It was suggested that LppQ could be used in a DIVA assay for discriminating animals vaccinated with the depleted T1LppQ-MT1 strain from those infected with the strains from the field [21, 22]. However, a genetically modified live attenuated vaccine might not be easily adopted by regulatory agencies, and there remains the disadvantages associated with a live attenuated vaccine including, the need for cold chain and possible side effects at the site of inoculation [23].

To develop a DIVA assay to be used with the newly developed CBPP subunit vaccines, four previously characterized antigens were used to test cattle sera to identify the most reliable antigen for identifying the infected and naïve animals. The four antigens (MSC_0397, MSC_0636, MSC_0653 and LppB) were selected based on extensive literature review and earlier laboratory work [4, 7, 8, 10, 12, 24]. Miltiadou et al.*,* [24] characterized LppB and predicted that it could be a possible antigen for use in a serological diagnostic test. Our study supports this prediction based on the high sensitivity and specificity observed. Although Lutta et al.*,* [14] showed that LppB detects chronically infected cattle, limited data were available to show that LppB could also detect the acute stages of CBPP.

Previously, Naseem et al.*,* [12] obtained 100% sensitivity and specificity in an ELISA test using both a conserved hypothetical (MSC_0636) and a glycosyltransferase (MSC_0108) *Mmm* proteins. The findings by Naseem et al.*,* [12] are in the range of those obtained in this study, sensitivities of MSC_0636 and LppB being 87.5% with both antigens showing specificities of 100%. The results by Naseem et al.*,* [12] using MSC_0636 and those obtained in this study seem to reach the same conclusions as those of Heller et al.*,* [8], who identified MSC_0136, MSC_0397, and MSC_0636 as the best-performing proteins in a cocktail iELISA. Heller *at al.,* [8] obtained sensitivity and specificity values of above 85.6 and 96.4% respectively in a cocktail ELISA using MSC_0136 and MSC_0636, which were similar to those obtained in our study. Although Naseem et al.*,* [12] reported sensitivity and specificity values of 100% using MSC_0108, the performance of this antigen was lower than MSC_0397 in the study by Heller et al.*,* [8] using a large number of serum samples. Therefore, we did not include MSC_0108 in our study. The results of our study show that MSC_0636 and LppB detected antibodies in sera for both acute and chronic stages of CBPP. Although we chose a relatively high cut-off (OD_405_ at ≤0.2) in our iELISA assays to increase specificity, the sensitivity was not affected as earlier reported [25]. It was also noted that all animals seroconverted by day 142.

We did not compare the same cattle sera used by previous authors and therefore, before the final validation of the DIVA diagnostic test proposed in our study, more cattle sera from field trials and from different time points after infection should be tested. A promising result is that the sensitivities and specificities obtained in this study are comparable to those of CFT and c-ELISA as prescribed by the OIE for the serological diagnosis of CBPP [26]. It was also noted that all antigens used in this study detected all positive sera collected at chronic stages of the disease, findings that correlate with earlier studies using LppB [14]. Early diagnosis and treatment of CBPP including the ability to detect chronic stages of the disease is important since it could accelerate CBPP control and/or eradication programs by testing, isolating, and/or treating infected cattle. Treatment prevents transmission of disease from carriers to naïve animals. Macrolides such as danofloxacin and more recently tulathromycin have been reported to reduce the spread of CBPP to healthy in-contact cattle that are treated at an appropriate time [27, 28].

Ten antigens (Table 2) were used to screen sera from the control group, subunit vaccinated, and CBPP-infected cattle. Amongst the ten antigens used in an iELISA platform in this study, MSC_0499, MSC_0776, MSC_0636, and LppB were determined as the most sensitive and specific antigens for a DIVA test. MSC_0499 and MSC_0776 were selected as the best performing vaccine proteins because the antigens detected vaccinated animals and showed very little reactivity with sera from infected animals, while MSC_0636 and LppB were selected as best performing non-vaccine proteins since the antigens detected infected animals and showed very little reactivity with sera from vaccinated animals. Our data suggest that MSC_0499 and MSC_0636 are the most sensitive and specific antigens for the development of serological assays for diagnosis and DIVA tests for CBPP.

## Conclusion

This study characterized ten *Mmm* antigens for use in a potential novel DIVA diagnostic for CBPP. The MSC_0499, MSC_0776, MSC_0636, and LppB proteins were able to differentiate cattle vaccinated with the subunit vaccine from those infected with a virulent *Mmm* Afade strain. Our preliminary analysis shows that *Mmm* antigens are potential targets for developing a DIVA diagnostic assay, though further testing of field sera from vaccinated and infected animals collected at different time intervals, and a cocktail made of the four antigens, should be undertaken to establish how useful a diagnostic test based on *Mmm* antigens will be.

## Methods

All protocols of this study were designed and performed in strict accordance as per applicable animal welfare regulations with the approval of KALRO-VSRI, Institutional Animal Care and Use Committee (IACUC): VSRI/IACUC009/15072016. Cattle owners were informed about the study before the purchase of cattle. There was no requirement for Vaccine and Infectious Disease Organization-International Vaccine Centre (VIDO-InterVac) to obtain ethical approval in Canada since the trials were conducted in Kenya.

### Sera from naive and CBPP infected cattle

Sera were obtained from the experiments described previously [29]. Thirty two serum samples were used in this study. Twenty-four of the sera were collected from eight male Zebu cattle (2 to 4 years old, weighing 105–214 kg) that had been infected with *Mmm* Afade and collected at different clinical stages: acute (3 weeks), subacute (7 weeks), and chronic (above 8 weeks) of the disease. From these eight infected animals at necropsy time, three had chronic sequestrae and the other five had consolidating acute lesions. *Mmm* was isolated from lung specimens of all the 8 CBPP-infected animals. The remaining serum samples were collected from eight male naïve zebu cattle (2 to 4 years old, weighing 105–214 kg). The sera from naïve cattle (sourced from Kakamega county, a CBPP negative area in Kenya) were well characterized and confirmed to be negative by both OIE prescribed serological tests, a CFT, and a c-ELISA.

### Sera from control group and subunit vaccinated cattle

Male Boran cattle (1 to 2 years old, weighing 249–390 kg) purchased from a CBPP free ranch, Kapiti Plains Estate Kenya were used in this study as previously described [7]. Briefly, using Microsoft Excel’s function, cattle were randomly assigned into three groups comprising 24 animals (Table 1). Eight of the animals from the unvaccinated group, were used as controls, while sixteen cattle were vaccinated with two test vaccines, each group consisting of eight animals (Table 1). All experimenters did not know the correspondence between the groups and treatments before the end of the trial and the blinding integrity was maintained throughout the study period. The vaccines used in this study (Table 1) were formulated at the VIDO-InterVac. Animals in each group were restrained in a cage, vaccinated twice subcutaneously using an 18-gauge needle with 2 ml of the vaccine formulation (Table 1), first on the left side of the neck on day 0, and the booster vaccine on the right side of the neck on day 28. Sampling was done on the following days: 0, 28, 49, 60, 81, 102, and 123. The blood samples were obtained from the jugular vein and collected into labeled BD Vacutainer® tubes (Becton, Dickson & Company, USA), then allowed to coagulate at room temperature for 2 h. The coagulated blood was centrifuged to separate the serum which was then aliquoted into Nunc® CryoTubes® (Sigma-Aldrich®, Germany). The serum samples were transported from the site of the trial (KALRO-VSRI) to Biosciences eastern and central Africa (BecA-ILRI) and stored at − 20^0^ C until further serological analysis to develop a DIVA test. Table 1 shows details of trial vaccines and controls. At 6 weeks post-challenge, cattle were euthanized by stunning with a captive bolt pistol and exsanguination. The carcasses were opened and lungs examined for CBPP lesions. No study animal entered the human food chain. The carcasses of all animals were disposed of in deep lime pits.

### Expression and purification of proteins for indirect ELISA

The genome sequences of non-vaccine antigens (MSC-0397, MSC_0636, and MSC-0653) were sent to GenScript USA Inc. for cloning into the pQE60 expression vector after which they were transformed into competent *E. coli* cells (BL 21 DE3 STAR). The strains containing these plasmids were kept at − 80^0^ C until use. The gene encoding LppB was cloned into pETite C-His Kan expression vector, transformed, and sent in glycerol stocks to BecA-ILRI from the Agricultural Research Council (ARC)-Onderstepoort Veterinary Institute (OVI), South Africa for expression and purification of antigens. The strains containing plasmids encoding the vaccine antigens (MSC_0136, MSC_0431, MSC_0499, and MSC_0775) were provided by the VIDO-InterVac, University of Saskatchewan, Canada. The agar slants were immediately streaked on agar plates containing 100 μg/ml ampicillin, incubated overnight, glycerol stocks prepared, and stored for future use. VIDO-InterVac also provided the MSC_0776 and MSC_0957 purified antigens.

The recombinant *Mmm* proteins were expressed as described with minor modifications [4]. Briefly, 250 ml of broth containing 100 μg/ml ampicillin was inoculated with 5 ml of the overnight culture in a 1-l flask and incubated at 37^0^ C in a shaker incubator at 220 rpm. The expression of proteins was induced in exponentially growing bacterial cultures (absorbance at 600 nm, 0.5–0.6) by the addition of 1 mM IPTG followed by incubation for 6 h at 37^0^ C at 220 rpm. The culture was then centrifuged in Beckman coulter Avanti J-301 at 8000 rpm for 15 min. The supernatant was discarded and pellets suspended in 1 ml of 25% sucrose/50 mM tris pH 8.0, transferred to 50 ml Corning tubes, and stored at − 80^0^ C until the day of purification. The LppB protein was expressed as earlier described [24].

The cell pellets were thawed for 15 min on ice and suspended in lysis buffer (with 8 M urea) at 10 ml per gram wet weight. 0.1 mM Phenyl methyl sulfonyl fluoride (PMSF)-Sigma Aldrich, USA was added to inhibit proteases. The suspended pellet was mixed for 60 min at room temperature (RT) taking care to avoid foaming. Recombinant proteins were purified by affinity chromatography on Nickel resin. The lysate was first centrifuged at 10, 000x g for 30 min at 15^0^ C to pellet the cellular debris and supernatant saved. 1 ml of a 50% Ni-NTA slurry (Sigma Aldrich, USA) was added to 10 ml lysate and mixed gently by shaking for 30 min at RT. The lysate-resin mixture was loaded into a column (BioRad, USA) and the flow-through collected for SDS-PAGE analysis. The columns were washed three times with 4 ml of wash buffer at pH 6.3 and wash fractions kept for SDS-PAGE analysis. Recombinant proteins were eluted three times with 1 ml elution buffer containing; 8 M urea, 0.1 M NaH_2_PO_4_, 1.5 M NaCl, 0.125 M imidazole at pH 7.4.

The purified proteins were extensively dialyzed in 4 M urea for 1.5 h then PBS for either 1 h (MSC_0499, MSC_0636, MSC_0653, and MSC_0775) or overnight at 4^0^ C (MSC_0136, MSC_0397, MSC_0431, and LppB) and concentrated using polyethylene glycol from Sigma Aldrich, Germany. Quantification was performed as described by the BCA Protein Assay Kit (Thermo Scientific, USA). Antigens (5 μl of each) were mixed with 2 μl 2x SDS sample buffer, and resolved in 10% SDS-PAGE gel.

### Characterization of the cattle immune responses by indirect ELISA

The protocol was adapted from Nkando et al.*,* [7] as follows: 96-well Nunc-Immuno-plate MaxiSorp; Thermo Fisher Scientific, USA were coated with 100 μl/well-containing 1 μg/ml of antigen in Na_2_CO_3_: NaHCO_3_ buffer (3.03 g Na_2_CO_3_; 6.0 g NaHCO_3_ in1000 ml dH_2_O for 100 mM at pH 9.6, diluted to 1:10 with dH_2_O before use) and incubated at 4^0^ C overnight. The following day, plates were washed five times with 300 μl wash buffer {PBS with 0.05% Tween 20 (PBST)} followed by blocking with 200 μl blocking buffer (PBST + 0.5% Horse serum from Gibco Life Technologies™, New Zealand) for 1 h in a shaker incubator at 37^0^ C. The wells were washed five times with 300 μl wash buffer before adding 100 μl serum samples (diluted 4-fold 1:100 in row A to 1: 409,600 in row G) followed by 1 h incubation at 37^0^ C and five washes as described above. In total, 100 μl alkaline phosphatase-conjugated rabbit-anti-bovine IgG antibody (KPL151–12-06, Sera Care, USA), diluted 1: 5000 in PBST, was added to each well before incubation for 1 h at 37^0^ C. Following five washes as above, 100 μl/well of para-Nitro phenyl phosphate (pNPP substrate, Sigma Aldrich, USA) was added. The reaction mixture was incubated for 45 min in the dark and the absorbance (OD) measured at 405 nm in a BioTek Synergy HT, USA plate reader.

### Determination of cut-off point, diagnostic sensitivity, and specificity of *Mmm* antigens

The iELISAs were done in duplicate and repeated on three different occasions. The negative and positive control sera were placed in duplicates in the first and last row of plates coated with 1 μg/ml of each antigen. No serum sample was placed in the second row and an OD of this row was subtracted from the OD containing sera. To analyze iELISA data based on *Mmm* antigens, the change-point method was determined as the cut-off point formula of a mean + 3 standard deviation of negative controls [30]. Percentage positivity (PP) was calculated as follows: PP = {(ODsample-OD-ve control)/ (OD + ve control-OD-ve control)} × 100% [31]. The standardized data was plotted in the box and scatter plots and cut-off OD values were used to discriminate the naïve, CBPP-infected, and subunit vaccinated animals. Sensitivity (Se), specificity (Sp), positive predictive value (PPV), and negative predictive value (NPV) were calculated as follows; Se: **{**TP/(TP + FN**)}** × 100%, where TP = true positive, FN = false negative; Sp: **{**TN/(TN + FP)**}** × 100%, where TN = true negative, FP = false positive; PPV: **{**TP/(TP + FP)**}** × 100%; NPV: **{**TN/(TN + FN)**}** × 100% [8, 32]. Sera from naïve and subunit vaccinated cattle were used to determine Se and Sp of *Mmm* vaccine antigens, while sera from naïve and CBPP infected cattle were used to determine Se and Sp of *Mmm* non-vaccine antigens. In this study, the number of animals per group (*n* = 8) was based on previous immunization trials [7, 33–36]. The resource equation method was used to show that our sample size was adequately powered to address the research question. Briefly, a value “E” = Total number of animals - Total number of groups; whereas in our study the “E” value for naïve group and CBPP-infected or control group and subunit-vaccinated cattle was determined as follows: {(8 × 2) - 2 = 14}. Our “E” value of 14 lied between 10 and 20, which is the acceptable limit [37, 38].

### Data analysis

Data for iELISA with different antigens were entered into MS Excel (Microsoft® Excel, Washington, 2016) and transferred into SPSS version 22, analyzed by two-way Analysis of Variance (ANOVA) and least significance difference used to separate the means. Data were imported into R software, version 3.6.0 (R Core Team, 2019) for analysis and drawing of the box and scatter plots. Cohen’s Kappa measurement assessed the level of agreement between the *Mmm* antigens.

## Supplementary information

**Additional file 1.** A comparison of the performance of vaccine and non-vaccine antigens. The SPSS was used to compare the performance of vaccine and non-vaccine antigens on sera from the control/naïve group, CBPP-infected, and subunit-vaccinated cattle.

**Additional file 2.** A comparison of the non-vaccine antigens on CBPP clinical stages. The SPSS was used to compare the performance of non-vaccine antigens on sera (collected at different clinical stages) from the naïve group and CBPP-infected cattle.

**Additional file 3. **ODs returned after testing sera from the control group and subunit-vaccinated cattle. The ODs were used to draw box and scatter plots, and determine the sensitivity and specificity of *Mmm* vaccine antigens.

**Additional file 4.** ODs returned after testing sera from naïve group and CBPP-infected cattle. The ODs were used to draw the box and scatter plots.

**Additional file 5. **ODs used to calculate the sensitivity and specificity of the naïve group and CBPP-infected cattle. The ODs were used to determine the sensitivity and specificity of *Mmm* non-vaccine antigens.

## Data Availability

The data on CBPP DIVA assay are the data used or analyzed in this paper and are available from the corresponding author (Harrison O. Lutta) on reasonable request. The datasets for CBPP subunit vaccines (International Patent Application No. PCT/CA2016/050864) are available on reasonable request from Dr. Andrew Potter, VIDO-InterVac.

## Abbreviations

ANOVA: Analysis of Variance
ARC: Agricultural Research Council
CBPP: Contagious Bovine Pleuropneumonia
CFT: Complement Fixation Test
DIVA: Differentiation of Infected from Vaccinated Animals
iELISA: Indirect Enzyme-Linked Immunosorbent Assay
ILRI: International Livestock Research Institute
KALRO: Kenya Agricultural & Livestock Research Organization
LppB: Lipoprotein B
LppQ: Lipoprotein Q
*Mmm*: *Mycoplasma mycoides* subsp. *mycoides*
NPV: Negative Predictive Value
OD: Optical density
OIE: Organization for Animal Health
OVI: Onderstepoort Veterinary Institute
PBS: Phosphate buffered saline
PCR: Polymerase Chain Reaction
PMSF: Phenyl Methyl Sulfonyl Fluoride
PPV: Positive Predictive Value
RT: Room Temperature
SDS-PAGE: Sodium Dodecyl Sulphate-Polyacrylamide Gel Electrophoresis
Se: Sensitivity
Sp: Specificity
VACNADA: Vaccines for Neglected Animal Diseases in Africa
VIDO-InterVac: Vaccine and Infectious Disease Organization-International Vaccine Centre
VSRI: Veterinary Science Research Institute.
