# Supplementary material for: Baseline analysis of Mycoplasma mycoides subsp. mycoides antigens as targets for a DIVA assay for use with a subunit vaccine for contagious bovine pleuropneumonia
Source: BMC Vet Res. 2020 Jul 10;16:236. doi: 10.1186/s12917-020-02453-w (PMC7350692; doi:10.1186/s12917-020-02453-w)
Supplement: Supplementary file 1 — Additional file 1. A comparison of the performance of vaccine and non-vaccine antigens. The SPSS was used to compare the performance of vaccine and non-vaccine antigens on sera from the control/naïve group, CBPP-infected, and subunit-vaccinated cattle. [file 12917_2020_2453_MOESM1_ESM.docx]

**Comparison of performance of vaccine and non-vaccine antigens**

**Univariate Analysis of Variance**

| **Notes** | | |
| --- | --- | --- |
| Output Created | | 21-AUG-2018 14:37:36 |
| Comments | |  |
| Input | Data | C:\Users\DNthiwa\OneDrive - CGIAR\Documents\mydata\Lutta data\coded_data_vaccines_control.xlsx.sav |
|  | Active Dataset | DataSet1 |
|  | Filter | <none> |
|  | Weight | <none> |
|  | Split File | antigen_type |
|  | N of Rows in Working Data File | 400 |
| Missing Value Handling | Definition of Missing | User-defined missing values are treated as missing. |
|  | Cases Used | Statistics are based on all cases with valid data for all variables in the model. |
| Syntax | | UNIANOVA optical_density BY protein_name  /METHOD=SSTYPE(3)  /INTERCEPT=INCLUDE  /POSTHOC=protein_name(LSD)  /CRITERIA=ALPHA(0.05)  /DESIGN=protein_name. |
| Resources | Processor Time | 00:00:00.00 |
|  | Elapsed Time | 00:00:00.02 |

**antigen_type = vacc_antigen**

| **Between-Subjects Factors^a^** | | | |
| --- | --- | --- | --- |
|  | | Value Label | N |
| protein_name | 1 | 136 | 48 |
|  | 2 | 431 | 48 |
|  | 3 | 499 | 48 |
|  | 4 | 775 | 32 |
|  | 9 | 776 | 16 |
|  | 10 | 957 | 16 |
| a. antigen_type = vacc_antigen | | | |

| **Tests of Between-Subjects Effects^a^** | | | | | |
| --- | --- | --- | --- | --- | --- |
| Dependent Variable: optical_density | | | | | |
| Source | Type III Sum of Squares | df | Mean Square | F | Sig. |
| Corrected Model | .665^b^ | 5 | .133 | 4.995 | .000 |
| Intercept | 3.973 | 1 | 3.973 | 149.246 | .000 |
| protein_name | .665 | 5 | .133 | 4.995 | .000 |
| Error | 5.378 | 202 | .027 |  |  |
| Total | 12.199 | 208 |  |  |  |
| Corrected Total | 6.043 | 207 |  |  |  |
| a. antigen_type = vacc_antigen | | | | | |
| b. R Squared = .110 (Adjusted R Squared = .088) | | | | | |

**Post Hoc Tests**

**protein_name**

| **Multiple Comparisons^a^** | | | | | | |
| --- | --- | --- | --- | --- | --- | --- |
| Dependent Variable: optical_density | | | | | | |
| LSD | | | | | | |
| (I) protein_name | (J) protein_name | Mean Difference (I-J) | Std. Error | Sig. | 95% Confidence Interval | |
|  |  |  |  |  | Lower Bound | Upper Bound |
| 136 | 431 | -.08931^*^ | .033305 | .008 | -.15498 | -.02364 |
|  | 499 | -.00056 | .033305 | .987 | -.06623 | .06511 |
|  | 775 | .07051 | .037237 | .060 | -.00291 | .14393 |
|  | 776 | .02842 | .047101 | .547 | -.06446 | .12129 |
|  | 957 | .08304 | .047101 | .079 | -.00983 | .17591 |
| 431 | 136 | .08931^*^ | .033305 | .008 | .02364 | .15498 |
|  | 499 | .08875^*^ | .033305 | .008 | .02308 | .15442 |
|  | 775 | .15982^*^ | .037237 | .000 | .08640 | .23325 |
|  | 776 | .11773^*^ | .047101 | .013 | .02486 | .21060 |
|  | 957 | .17235^*^ | .047101 | .000 | .07948 | .26523 |
| 499 | 136 | .00056 | .033305 | .987 | -.06511 | .06623 |
|  | 431 | -.08875^*^ | .033305 | .008 | -.15442 | -.02308 |
|  | 775 | .07107 | .037237 | .058 | -.00235 | .14450 |
|  | 776 | .02898 | .047101 | .539 | -.06389 | .12185 |
|  | 957 | .08360 | .047101 | .077 | -.00927 | .17648 |
| 775 | 136 | -.07051 | .037237 | .060 | -.14393 | .00291 |
|  | 431 | -.15982^*^ | .037237 | .000 | -.23325 | -.08640 |
|  | 499 | -.07107 | .037237 | .058 | -.14450 | .00235 |
|  | 776 | -.04209 | .049958 | .400 | -.14060 | .05641 |
|  | 957 | .01253 | .049958 | .802 | -.08598 | .11104 |
| 776 | 136 | -.02842 | .047101 | .547 | -.12129 | .06446 |
|  | 431 | -.11773^*^ | .047101 | .013 | -.21060 | -.02486 |
|  | 499 | -.02898 | .047101 | .539 | -.12185 | .06389 |
|  | 775 | .04209 | .049958 | .400 | -.05641 | .14060 |
|  | 957 | .05462 | .057687 | .345 | -.05912 | .16837 |
| 957 | 136 | -.08304 | .047101 | .079 | -.17591 | .00983 |
|  | 431 | -.17235^*^ | .047101 | .000 | -.26523 | -.07948 |
|  | 499 | -.08360 | .047101 | .077 | -.17648 | .00927 |
|  | 775 | -.01253 | .049958 | .802 | -.11104 | .08598 |
|  | 776 | -.05462 | .057687 | .345 | -.16837 | .05912 |
| Based on observed means.  The error term is Mean Square(Error) = .027.^a^ | | | | | | |
| *. The mean difference is significant at the 0.05 level. | | | | | | |
| a. antigen_type = vacc_antigen | | | | | | |

**Homogeneous Subsets**

| **optical_density^a^** |
| --- |
|  |
| Means for groups in homogeneous subsets are displayed.  Based on observed means.  The error term is Mean Square(Error) = .027.^a^ |
| a. antigen_type = vacc_antigen |

**antigen_type = non_vacc_antigen**

| **Between-Subjects Factors^a^** | | | |
| --- | --- | --- | --- |
|  | | Value Label | N |
| protein_name | 5 | 397 | 48 |
|  | 6 | 636 | 48 |
|  | 7 | 653 | 48 |
|  | 8 | LppB | 48 |
| a. antigen_type = non_vacc_antigen | | | |

| **Tests of Between-Subjects Effects^a^** | | | | | |
| --- | --- | --- | --- | --- | --- |
| Dependent Variable: optical_density | | | | | |
| Source | Type III Sum of Squares | df | Mean Square | F | Sig. |
| Corrected Model | .353^b^ | 3 | .118 | 4.152 | .007 |
| Intercept | 3.405 | 1 | 3.405 | 120.143 | .000 |
| protein_name | .353 | 3 | .118 | 4.152 | .007 |
| Error | 5.327 | 188 | .028 |  |  |
| Total | 9.085 | 192 |  |  |  |
| Corrected Total | 5.680 | 191 |  |  |  |
| a. antigen_type = non_vacc_antigen | | | | | |
| b. R Squared = .062 (Adjusted R Squared = .047) | | | | | |

**Post Hoc Tests**

**protein_name**

| **Multiple Comparisons^a^** | | | | | | |
| --- | --- | --- | --- | --- | --- | --- |
| Dependent Variable: optical_density | | | | | | |
| LSD | | | | | | |
| (I) protein_name | (J) protein_name | Mean Difference (I-J) | Std. Error | Sig. | 95% Confidence Interval | |
|  |  |  |  |  | Lower Bound | Upper Bound |
| 397 | 636 | .00638 | .034362 | .853 | -.06141 | .07416 |
|  | 653 | -.09752^*^ | .034362 | .005 | -.16530 | -.02974 |
|  | LppB | -.00275 | .034362 | .936 | -.07053 | .06503 |
| 636 | 397 | -.00638 | .034362 | .853 | -.07416 | .06141 |
|  | 653 | -.10390^*^ | .034362 | .003 | -.17168 | -.03611 |
|  | LppB | -.00912 | .034362 | .791 | -.07691 | .05866 |
| 653 | 397 | .09752^*^ | .034362 | .005 | .02974 | .16530 |
|  | 636 | .10390^*^ | .034362 | .003 | .03611 | .17168 |
|  | LppB | .09477^*^ | .034362 | .006 | .02699 | .16255 |
| LppB | 397 | .00275 | .034362 | .936 | -.06503 | .07053 |
|  | 636 | .00912 | .034362 | .791 | -.05866 | .07691 |
|  | 653 | -.09477^*^ | .034362 | .006 | -.16255 | -.02699 |
| Based on observed means.  The error term is Mean Square(Error) = .028.^a^ | | | | | | |
| *. The mean difference is significant at the 0.05 level. | | | | | | |
| a. antigen_type = non_vacc_antigen | | | | | | |

**Homogeneous Subsets**

| **optical_density^a^** |
| --- |
|  |
| Means for groups in homogeneous subsets are displayed.  Based on observed means.  The error term is Mean Square(Error) = .028.^a^ |
| a. antigen_type = non_vacc_antigen |

SORT CASES BY group_type.

SPLIT FILE SEPARATE BY group_type.

UNIANOVA optical_density BY protein_name

/METHOD=SSTYPE(3)

/INTERCEPT=INCLUDE

/POSTHOC=protein_name(LSD)

/CRITERIA=ALPHA(0.05)

/DESIGN=protein_name.

**Univariate Analysis of Variance**

| **Notes** | | |
| --- | --- | --- |
| Output Created | | 21-AUG-2018 14:38:46 |
| Comments | |  |
| Input | Data | C:\Users\DNthiwa\OneDrive - CGIAR\Documents\mydata\Lutta data\coded_data_vaccines_control.xlsx.sav |
|  | Active Dataset | DataSet1 |
|  | Filter | <none> |
|  | Weight | <none> |
|  | Split File | group_type |
|  | N of Rows in Working Data File | 400 |
| Missing Value Handling | Definition of Missing | User-defined missing values are treated as missing. |
|  | Cases Used | Statistics are based on all cases with valid data for all variables in the model. |
| Syntax | | UNIANOVA optical_density BY protein_name  /METHOD=SSTYPE(3)  /INTERCEPT=INCLUDE  /POSTHOC=protein_name(LSD)  /CRITERIA=ALPHA(0.05)  /DESIGN=protein_name. |
| Resources | Processor Time | 00:00:00.02 |
|  | Elapsed Time | 00:00:00.03 |

**group_type = vacc_A_control**

| **Between-Subjects Factors^a^** | | | |
| --- | --- | --- | --- |
|  | | Value Label | N |
| protein_name | 1 | 136 | 16 |
|  | 2 | 431 | 16 |
|  | 3 | 499 | 16 |
|  | 4 | 775 | 16 |
|  | 5 | 397 | 16 |
|  | 6 | 636 | 16 |
|  | 7 | 653 | 16 |
|  | 8 | LppB | 16 |
| a. group_type = vacc_A_control | | | |

| **Tests of Between-Subjects Effects^a^** | | | | | |
| --- | --- | --- | --- | --- | --- |
| Dependent Variable: optical_density | | | | | |
| Source | Type III Sum of Squares | df | Mean Square | F | Sig. |
| Corrected Model | .009^b^ | 7 | .001 | 1.852 | .084 |
| Intercept | 1.348 | 1 | 1.348 | 1900.668 | .000 |
| protein_name | .009 | 7 | .001 | 1.852 | .084 |
| Error | .085 | 120 | .001 |  |  |
| Total | 1.443 | 128 |  |  |  |
| Corrected Total | .094 | 127 |  |  |  |
| a. group_type = vacc_A_control | | | | | |
| b. R Squared = .097 (Adjusted R Squared = .045) | | | | | |

**Post Hoc Tests**

**protein_name**

| **Multiple Comparisons^a^** | | | | | | |
| --- | --- | --- | --- | --- | --- | --- |
| Dependent Variable: optical_density | | | | | | |
| LSD | | | | | | |
| (I) protein_name | (J) protein_name | Mean Difference (I-J) | Std. Error | Sig. | 95% Confidence Interval | |
|  |  |  |  |  | Lower Bound | Upper Bound |
| 136 | 431 | .02612^*^ | .009417 | .006 | .00748 | .04477 |
|  | 499 | .01794 | .009417 | .059 | -.00071 | .03658 |
|  | 775 | .02400^*^ | .009417 | .012 | .00535 | .04265 |
|  | 397 | .01344 | .009417 | .156 | -.00521 | .03208 |
|  | 636 | .01919^*^ | .009417 | .044 | .00054 | .03783 |
|  | 653 | .00550 | .009417 | .560 | -.01315 | .02415 |
|  | LppB | .00969 | .009417 | .306 | -.00896 | .02833 |
| 431 | 136 | -.02612^*^ | .009417 | .006 | -.04477 | -.00748 |
|  | 499 | -.00819 | .009417 | .386 | -.02683 | .01046 |
|  | 775 | -.00213 | .009417 | .822 | -.02077 | .01652 |
|  | 397 | -.01269 | .009417 | .180 | -.03133 | .00596 |
|  | 636 | -.00694 | .009417 | .463 | -.02558 | .01171 |
|  | 653 | -.02063^*^ | .009417 | .030 | -.03927 | -.00198 |
|  | LppB | -.01644 | .009417 | .083 | -.03508 | .00221 |
| 499 | 136 | -.01794 | .009417 | .059 | -.03658 | .00071 |
|  | 431 | .00819 | .009417 | .386 | -.01046 | .02683 |
|  | 775 | .00606 | .009417 | .521 | -.01258 | .02471 |
|  | 397 | -.00450 | .009417 | .634 | -.02315 | .01415 |
|  | 636 | .00125 | .009417 | .895 | -.01740 | .01990 |
|  | 653 | -.01244 | .009417 | .189 | -.03108 | .00621 |
|  | LppB | -.00825 | .009417 | .383 | -.02690 | .01040 |
| 775 | 136 | -.02400^*^ | .009417 | .012 | -.04265 | -.00535 |
|  | 431 | .00213 | .009417 | .822 | -.01652 | .02077 |
|  | 499 | -.00606 | .009417 | .521 | -.02471 | .01258 |
|  | 397 | -.01056 | .009417 | .264 | -.02921 | .00808 |
|  | 636 | -.00481 | .009417 | .610 | -.02346 | .01383 |
|  | 653 | -.01850 | .009417 | .052 | -.03715 | .00015 |
|  | LppB | -.01431 | .009417 | .131 | -.03296 | .00433 |
| 397 | 136 | -.01344 | .009417 | .156 | -.03208 | .00521 |
|  | 431 | .01269 | .009417 | .180 | -.00596 | .03133 |
|  | 499 | .00450 | .009417 | .634 | -.01415 | .02315 |
|  | 775 | .01056 | .009417 | .264 | -.00808 | .02921 |
|  | 636 | .00575 | .009417 | .543 | -.01290 | .02440 |
|  | 653 | -.00794 | .009417 | .401 | -.02658 | .01071 |
|  | LppB | -.00375 | .009417 | .691 | -.02240 | .01490 |
| 636 | 136 | -.01919^*^ | .009417 | .044 | -.03783 | -.00054 |
|  | 431 | .00694 | .009417 | .463 | -.01171 | .02558 |
|  | 499 | -.00125 | .009417 | .895 | -.01990 | .01740 |
|  | 775 | .00481 | .009417 | .610 | -.01383 | .02346 |
|  | 397 | -.00575 | .009417 | .543 | -.02440 | .01290 |
|  | 653 | -.01369 | .009417 | .149 | -.03233 | .00496 |
|  | LppB | -.00950 | .009417 | .315 | -.02815 | .00915 |
| 653 | 136 | -.00550 | .009417 | .560 | -.02415 | .01315 |
|  | 431 | .02063^*^ | .009417 | .030 | .00198 | .03927 |
|  | 499 | .01244 | .009417 | .189 | -.00621 | .03108 |
|  | 775 | .01850 | .009417 | .052 | -.00015 | .03715 |
|  | 397 | .00794 | .009417 | .401 | -.01071 | .02658 |
|  | 636 | .01369 | .009417 | .149 | -.00496 | .03233 |
|  | LppB | .00419 | .009417 | .657 | -.01446 | .02283 |
| LppB | 136 | -.00969 | .009417 | .306 | -.02833 | .00896 |
|  | 431 | .01644 | .009417 | .083 | -.00221 | .03508 |
|  | 499 | .00825 | .009417 | .383 | -.01040 | .02690 |
|  | 775 | .01431 | .009417 | .131 | -.00433 | .03296 |
|  | 397 | .00375 | .009417 | .691 | -.01490 | .02240 |
|  | 636 | .00950 | .009417 | .315 | -.00915 | .02815 |
|  | 653 | -.00419 | .009417 | .657 | -.02283 | .01446 |
| Based on observed means.  The error term is Mean Square(Error) = .001.^a^ | | | | | | |
| *. The mean difference is significant at the 0.05 level. | | | | | | |
| a. group_type = vacc_A_control | | | | | | |

**Homogeneous Subsets**

| **optical_density^a^** |
| --- |
|  |
| Means for groups in homogeneous subsets are displayed.  Based on observed means.  The error term is Mean Square(Error) = .001.^a^ |
| a. group_type = vacc_A_control |

**group_type = vaccine_B**

| **Between-Subjects Factors^a^** | | | |
| --- | --- | --- | --- |
|  | | Value Label | N |
| protein_name | 1 | 136 | 16 |
|  | 2 | 431 | 16 |
|  | 3 | 499 | 16 |
|  | 4 | 775 | 16 |
|  | 5 | 397 | 16 |
|  | 6 | 636 | 16 |
|  | 7 | 653 | 16 |
|  | 8 | LppB | 16 |
| a. group_type = vaccine_B | | | |

| **Tests of Between-Subjects Effects^a^** | | | | | |
| --- | --- | --- | --- | --- | --- |
| Dependent Variable: optical_density | | | | | |
| Source | Type III Sum of Squares | df | Mean Square | F | Sig. |
| Corrected Model | 1.181^b^ | 7 | .169 | 9.963 | .000 |
| Intercept | 2.851 | 1 | 2.851 | 168.391 | .000 |
| protein_name | 1.181 | 7 | .169 | 9.963 | .000 |
| Error | 2.031 | 120 | .017 |  |  |
| Total | 6.063 | 128 |  |  |  |
| Corrected Total | 3.212 | 127 |  |  |  |
| a. group_type = vaccine_B | | | | | |
| b. R Squared = .368 (Adjusted R Squared = .331) | | | | | |

**Post Hoc Tests**

**protein_name**

| **Multiple Comparisons^a^** | | | | | | |
| --- | --- | --- | --- | --- | --- | --- |
| Dependent Variable: optical_density | | | | | | |
| LSD | | | | | | |
| (I) protein_name | (J) protein_name | Mean Difference (I-J) | Std. Error | Sig. | 95% Confidence Interval | |
|  |  |  |  |  | Lower Bound | Upper Bound |
| 136 | 431 | -.27294^*^ | .046001 | .000 | -.36402 | -.18186 |
|  | 499 | .01869 | .046001 | .685 | -.07239 | .10977 |
|  | 775 | .02206 | .046001 | .632 | -.06902 | .11314 |
|  | 397 | .02381 | .046001 | .606 | -.06727 | .11489 |
|  | 636 | .02425 | .046001 | .599 | -.06683 | .11533 |
|  | 653 | .00419 | .046001 | .928 | -.08689 | .09527 |
|  | LppB | .02106 | .046001 | .648 | -.07002 | .11214 |
| 431 | 136 | .27294^*^ | .046001 | .000 | .18186 | .36402 |
|  | 499 | .29162^*^ | .046001 | .000 | .20055 | .38270 |
|  | 775 | .29500^*^ | .046001 | .000 | .20392 | .38608 |
|  | 397 | .29675^*^ | .046001 | .000 | .20567 | .38783 |
|  | 636 | .29719^*^ | .046001 | .000 | .20611 | .38827 |
|  | 653 | .27712^*^ | .046001 | .000 | .18605 | .36820 |
|  | LppB | .29400^*^ | .046001 | .000 | .20292 | .38508 |
| 499 | 136 | -.01869 | .046001 | .685 | -.10977 | .07239 |
|  | 431 | -.29162^*^ | .046001 | .000 | -.38270 | -.20055 |
|  | 775 | .00338 | .046001 | .942 | -.08770 | .09445 |
|  | 397 | .00513 | .046001 | .911 | -.08595 | .09620 |
|  | 636 | .00556 | .046001 | .904 | -.08552 | .09664 |
|  | 653 | -.01450 | .046001 | .753 | -.10558 | .07658 |
|  | LppB | .00238 | .046001 | .959 | -.08870 | .09345 |
| 775 | 136 | -.02206 | .046001 | .632 | -.11314 | .06902 |
|  | 431 | -.29500^*^ | .046001 | .000 | -.38608 | -.20392 |
|  | 499 | -.00338 | .046001 | .942 | -.09445 | .08770 |
|  | 397 | .00175 | .046001 | .970 | -.08933 | .09283 |
|  | 636 | .00219 | .046001 | .962 | -.08889 | .09327 |
|  | 653 | -.01788 | .046001 | .698 | -.10895 | .07320 |
|  | LppB | -.00100 | .046001 | .983 | -.09208 | .09008 |
| 397 | 136 | -.02381 | .046001 | .606 | -.11489 | .06727 |
|  | 431 | -.29675^*^ | .046001 | .000 | -.38783 | -.20567 |
|  | 499 | -.00513 | .046001 | .911 | -.09620 | .08595 |
|  | 775 | -.00175 | .046001 | .970 | -.09283 | .08933 |
|  | 636 | .00044 | .046001 | .992 | -.09064 | .09152 |
|  | 653 | -.01963 | .046001 | .670 | -.11070 | .07145 |
|  | LppB | -.00275 | .046001 | .952 | -.09383 | .08833 |
| 636 | 136 | -.02425 | .046001 | .599 | -.11533 | .06683 |
|  | 431 | -.29719^*^ | .046001 | .000 | -.38827 | -.20611 |
|  | 499 | -.00556 | .046001 | .904 | -.09664 | .08552 |
|  | 775 | -.00219 | .046001 | .962 | -.09327 | .08889 |
|  | 397 | -.00044 | .046001 | .992 | -.09152 | .09064 |
|  | 653 | -.02006 | .046001 | .664 | -.11114 | .07102 |
|  | LppB | -.00319 | .046001 | .945 | -.09427 | .08789 |
| 653 | 136 | -.00419 | .046001 | .928 | -.09527 | .08689 |
|  | 431 | -.27712^*^ | .046001 | .000 | -.36820 | -.18605 |
|  | 499 | .01450 | .046001 | .753 | -.07658 | .10558 |
|  | 775 | .01788 | .046001 | .698 | -.07320 | .10895 |
|  | 397 | .01963 | .046001 | .670 | -.07145 | .11070 |
|  | 636 | .02006 | .046001 | .664 | -.07102 | .11114 |
|  | LppB | .01688 | .046001 | .714 | -.07420 | .10795 |
| LppB | 136 | -.02106 | .046001 | .648 | -.11214 | .07002 |
|  | 431 | -.29400^*^ | .046001 | .000 | -.38508 | -.20292 |
|  | 499 | -.00238 | .046001 | .959 | -.09345 | .08870 |
|  | 775 | .00100 | .046001 | .983 | -.09008 | .09208 |
|  | 397 | .00275 | .046001 | .952 | -.08833 | .09383 |
|  | 636 | .00319 | .046001 | .945 | -.08789 | .09427 |
|  | 653 | -.01688 | .046001 | .714 | -.10795 | .07420 |
| Based on observed means.  The error term is Mean Square(Error) = .017.^a^ | | | | | | |
| *. The mean difference is significant at the 0.05 level. | | | | | | |
| a. group_type = vaccine_B | | | | | | |

**Homogeneous Subsets**

| **optical_density^a^** |
| --- |
|  |
| Means for groups in homogeneous subsets are displayed.  Based on observed means.  The error term is Mean Square(Error) = .017.^a^ |
| a. group_type = vaccine_B |

**group_type = vaccine_C**

| **Between-Subjects Factors^a^** | | | |
| --- | --- | --- | --- |
|  | | Value Label | N |
| protein_name | 1 | 136 | 16 |
|  | 2 | 431 | 16 |
|  | 3 | 499 | 16 |
|  | 5 | 397 | 16 |
|  | 6 | 636 | 16 |
|  | 7 | 653 | 16 |
|  | 8 | LppB | 16 |
|  | 9 | 776 | 16 |
|  | 10 | 957 | 16 |
| a. group_type = vaccine_C | | | |

| **Tests of Between-Subjects Effects^a^** | | | | | |
| --- | --- | --- | --- | --- | --- |
| Dependent Variable: optical_density | | | | | |
| Source | Type III Sum of Squares | df | Mean Square | F | Sig. |
| Corrected Model | 1.504^b^ | 8 | .188 | 3.971 | .000 |
| Intercept | 5.886 | 1 | 5.886 | 124.364 | .000 |
| protein_name | 1.504 | 8 | .188 | 3.971 | .000 |
| Error | 6.389 | 135 | .047 |  |  |
| Total | 13.779 | 144 |  |  |  |
| Corrected Total | 7.893 | 143 |  |  |  |
| a. group_type = vaccine_C | | | | | |
| b. R Squared = .191 (Adjusted R Squared = .143) | | | | | |

**Post Hoc Tests**

**protein_name**

| **Multiple Comparisons^a^** | | | | | | |
| --- | --- | --- | --- | --- | --- | --- |
| Dependent Variable: optical_density | | | | | | |
| LSD | | | | | | |
| (I) protein_name | (J) protein_name | Mean Difference (I-J) | Std. Error | Sig. | 95% Confidence Interval | |
|  |  |  |  |  | Lower Bound | Upper Bound |
| 136 | 431 | -.02112 | .076916 | .784 | -.17324 | .13099 |
|  | 499 | -.03831 | .076916 | .619 | -.19043 | .11380 |
|  | 397 | .14588 | .076916 | .060 | -.00624 | .29799 |
|  | 636 | .15881^*^ | .076916 | .041 | .00670 | .31093 |
|  | 653 | -.11912 | .076916 | .124 | -.27124 | .03299 |
|  | LppB | .14413 | .076916 | .063 | -.00799 | .29624 |
|  | 776 | .12338 | .076916 | .111 | -.02874 | .27549 |
|  | 957 | .17800^*^ | .076916 | .022 | .02588 | .33012 |
| 431 | 136 | .02112 | .076916 | .784 | -.13099 | .17324 |
|  | 499 | -.01719 | .076916 | .824 | -.16930 | .13493 |
|  | 397 | .16700^*^ | .076916 | .032 | .01488 | .31912 |
|  | 636 | .17994^*^ | .076916 | .021 | .02782 | .33205 |
|  | 653 | -.09800 | .076916 | .205 | -.25012 | .05412 |
|  | LppB | .16525^*^ | .076916 | .033 | .01313 | .31737 |
|  | 776 | .14450 | .076916 | .062 | -.00762 | .29662 |
|  | 957 | .19912^*^ | .076916 | .011 | .04701 | .35124 |
| 499 | 136 | .03831 | .076916 | .619 | -.11380 | .19043 |
|  | 431 | .01719 | .076916 | .824 | -.13493 | .16930 |
|  | 397 | .18419^*^ | .076916 | .018 | .03207 | .33630 |
|  | 636 | .19713^*^ | .076916 | .011 | .04501 | .34924 |
|  | 653 | -.08081 | .076916 | .295 | -.23293 | .07130 |
|  | LppB | .18244^*^ | .076916 | .019 | .03032 | .33455 |
|  | 776 | .16169^*^ | .076916 | .037 | .00957 | .31380 |
|  | 957 | .21631^*^ | .076916 | .006 | .06420 | .36843 |
| 397 | 136 | -.14588 | .076916 | .060 | -.29799 | .00624 |
|  | 431 | -.16700^*^ | .076916 | .032 | -.31912 | -.01488 |
|  | 499 | -.18419^*^ | .076916 | .018 | -.33630 | -.03207 |
|  | 636 | .01294 | .076916 | .867 | -.13918 | .16505 |
|  | 653 | -.26500^*^ | .076916 | .001 | -.41712 | -.11288 |
|  | LppB | -.00175 | .076916 | .982 | -.15387 | .15037 |
|  | 776 | -.02250 | .076916 | .770 | -.17462 | .12962 |
|  | 957 | .03213 | .076916 | .677 | -.11999 | .18424 |
| 636 | 136 | -.15881^*^ | .076916 | .041 | -.31093 | -.00670 |
|  | 431 | -.17994^*^ | .076916 | .021 | -.33205 | -.02782 |
|  | 499 | -.19713^*^ | .076916 | .011 | -.34924 | -.04501 |
|  | 397 | -.01294 | .076916 | .867 | -.16505 | .13918 |
|  | 653 | -.27794^*^ | .076916 | .000 | -.43005 | -.12582 |
|  | LppB | -.01469 | .076916 | .849 | -.16680 | .13743 |
|  | 776 | -.03544 | .076916 | .646 | -.18755 | .11668 |
|  | 957 | .01919 | .076916 | .803 | -.13293 | .17130 |
| 653 | 136 | .11912 | .076916 | .124 | -.03299 | .27124 |
|  | 431 | .09800 | .076916 | .205 | -.05412 | .25012 |
|  | 499 | .08081 | .076916 | .295 | -.07130 | .23293 |
|  | 397 | .26500^*^ | .076916 | .001 | .11288 | .41712 |
|  | 636 | .27794^*^ | .076916 | .000 | .12582 | .43005 |
|  | LppB | .26325^*^ | .076916 | .001 | .11113 | .41537 |
|  | 776 | .24250^*^ | .076916 | .002 | .09038 | .39462 |
|  | 957 | .29713^*^ | .076916 | .000 | .14501 | .44924 |
| LppB | 136 | -.14413 | .076916 | .063 | -.29624 | .00799 |
|  | 431 | -.16525^*^ | .076916 | .033 | -.31737 | -.01313 |
|  | 499 | -.18244^*^ | .076916 | .019 | -.33455 | -.03032 |
|  | 397 | .00175 | .076916 | .982 | -.15037 | .15387 |
|  | 636 | .01469 | .076916 | .849 | -.13743 | .16680 |
|  | 653 | -.26325^*^ | .076916 | .001 | -.41537 | -.11113 |
|  | 776 | -.02075 | .076916 | .788 | -.17287 | .13137 |
|  | 957 | .03388 | .076916 | .660 | -.11824 | .18599 |
| 776 | 136 | -.12338 | .076916 | .111 | -.27549 | .02874 |
|  | 431 | -.14450 | .076916 | .062 | -.29662 | .00762 |
|  | 499 | -.16169^*^ | .076916 | .037 | -.31380 | -.00957 |
|  | 397 | .02250 | .076916 | .770 | -.12962 | .17462 |
|  | 636 | .03544 | .076916 | .646 | -.11668 | .18755 |
|  | 653 | -.24250^*^ | .076916 | .002 | -.39462 | -.09038 |
|  | LppB | .02075 | .076916 | .788 | -.13137 | .17287 |
|  | 957 | .05462 | .076916 | .479 | -.09749 | .20674 |
| 957 | 136 | -.17800^*^ | .076916 | .022 | -.33012 | -.02588 |
|  | 431 | -.19912^*^ | .076916 | .011 | -.35124 | -.04701 |
|  | 499 | -.21631^*^ | .076916 | .006 | -.36843 | -.06420 |
|  | 397 | -.03213 | .076916 | .677 | -.18424 | .11999 |
|  | 636 | -.01919 | .076916 | .803 | -.17130 | .13293 |
|  | 653 | -.29713^*^ | .076916 | .000 | -.44924 | -.14501 |
|  | LppB | -.03388 | .076916 | .660 | -.18599 | .11824 |
|  | 776 | -.05462 | .076916 | .479 | -.20674 | .09749 |
| Based on observed means.  The error term is Mean Square(Error) = .047.^a^ | | | | | | |
| *. The mean difference is significant at the 0.05 level. | | | | | | |
| a. group_type = vaccine_C | | | | | | |

**Homogeneous Subsets**

| **optical_density^a^** |
| --- |
|  |
| Means for groups in homogeneous subsets are displayed.  Based on observed means.  The error term is Mean Square(Error) = .047.^a^ |
| a. group_type = vaccine_C |
