# Supplementary material for: Baseline analysis of Mycoplasma mycoides subsp. mycoides antigens as targets for a DIVA assay for use with a subunit vaccine for contagious bovine pleuropneumonia
Source: BMC Vet Res. 2020 Jul 10;16:236. doi: 10.1186/s12917-020-02453-w (PMC7350692; doi:10.1186/s12917-020-02453-w)
Supplement: Supplementary file 2 — Additional file 2. A comparison of the non-vaccine antigens on CBPP clinical stages. The SPSS was used to compare the performance of non-vaccine antigens on sera (collected at different clinical stages) from the naïve group and CBPP-infected cattle. [file 12917_2020_2453_MOESM2_ESM.docx]

**Comparison of non-vaccine antigens on CBPP clinical stages**

**Univariate Analysis of Variance**

| **Notes** | | |
| --- | --- | --- |
| Output Created | | 30-JUL-2018 11:10:13 |
| Comments | |  |
| Input | Data | C:\Users\DNthiwa\OneDrive - CGIAR\Documents\mydata\Lutta data\lutta_data.sav |
|  | Active Dataset | DataSet2 |
|  | Filter | <none> |
|  | Weight | <none> |
|  | Split File | <none> |
|  | N of Rows in Working Data File | 128 |
| Missing Value Handling | Definition of Missing | User-defined missing values are treated as missing. |
|  | Cases Used | Statistics are based on all cases with valid data for all variables in the model. |
| Syntax | | UNIANOVA optical_density BY disease_stage  /METHOD=SSTYPE(3)  /INTERCEPT=INCLUDE  /CRITERIA=ALPHA(0.05)  /DESIGN=disease_stage. |
| Resources | Processor Time | 00:00:00.02 |
|  | Elapsed Time | 00:00:00.02 |

| **Between-Subjects Factors** | | | |
| --- | --- | --- | --- |
|  | | Value Label | N |
| disease_stage | 1 | acute | 32 |
|  | 2 | sub_acute | 32 |
|  | 3 | chronic | 32 |

| **Tests of Between-Subjects Effects** | | | | | |
| --- | --- | --- | --- | --- | --- |
| Dependent Variable: optical_density | | | | | |
| Source | Type III Sum of Squares | df | Mean Square | F | Sig. |
| Corrected Model | .295^a^ | 2 | .148 | 9.154 | .000 |
| Intercept | 5.796 | 1 | 5.796 | 359.147 | .000 |
| disease_stage | .295 | 2 | .148 | 9.154 | .000 |
| Error | 1.501 | 93 | .016 |  |  |
| Total | 7.593 | 96 |  |  |  |
| Corrected Total | 1.796 | 95 |  |  |  |
| a. R Squared = .164 (Adjusted R Squared = .147) | | | | | |

UNIANOVA optical_density BY disease_stage

/METHOD=SSTYPE(3)

/INTERCEPT=INCLUDE

/CRITERIA=ALPHA(0.05)

/DESIGN=disease_stage.

UNIANOVA optical_density BY disease_stage

/METHOD=SSTYPE(3)

/INTERCEPT=INCLUDE

/POSTHOC=disease_stage(LSD)

/CRITERIA=ALPHA(0.05)

/DESIGN=disease_stage.

**Univariate Analysis of Variance**

| **Notes** | | |
| --- | --- | --- |
| Output Created | | 30-JUL-2018 11:11:38 |
| Comments | |  |
| Input | Data | C:\Users\DNthiwa\OneDrive - CGIAR\Documents\mydata\Lutta data\lutta_data.sav |
|  | Active Dataset | DataSet2 |
|  | Filter | <none> |
|  | Weight | <none> |
|  | Split File | <none> |
|  | N of Rows in Working Data File | 128 |
| Missing Value Handling | Definition of Missing | User-defined missing values are treated as missing. |
|  | Cases Used | Statistics are based on all cases with valid data for all variables in the model. |
| Syntax | | UNIANOVA optical_density BY disease_stage  /METHOD=SSTYPE(3)  /INTERCEPT=INCLUDE  /POSTHOC=disease_stage(LSD)  /CRITERIA=ALPHA(0.05)  /DESIGN=disease_stage. |
| Resources | Processor Time | 00:00:00.00 |
|  | Elapsed Time | 00:00:00.00 |

| **Between-Subjects Factors** | | | |
| --- | --- | --- | --- |
|  | | Value Label | N |
| disease_stage | 1 | acute | 32 |
|  | 2 | sub_acute | 32 |
|  | 3 | chronic | 32 |

| **Tests of Between-Subjects Effects** | | | | | |
| --- | --- | --- | --- | --- | --- |
| Dependent Variable: optical_density | | | | | |
| Source | Type III Sum of Squares | df | Mean Square | F | Sig. |
| Corrected Model | .295^a^ | 2 | .148 | 9.154 | .000 |
| Intercept | 5.796 | 1 | 5.796 | 359.147 | .000 |
| disease_stage | .295 | 2 | .148 | 9.154 | .000 |
| Error | 1.501 | 93 | .016 |  |  |
| Total | 7.593 | 96 |  |  |  |
| Corrected Total | 1.796 | 95 |  |  |  |
| a. R Squared = .164 (Adjusted R Squared = .147) | | | | | |

**Post Hoc Tests**

**disease_stage**

| **Multiple Comparisons** | | | | | | |
| --- | --- | --- | --- | --- | --- | --- |
| Dependent Variable: optical_density | | | | | | |
| LSD | | | | | | |
| (I) disease_stage | (J) disease_stage | Mean Difference (I-J) | Std. Error | Sig. | 95% Confidence Interval | |
|  |  |  |  |  | Lower Bound | Upper Bound |
| acute | sub_acute | .02119 | .031760 | .506 | -.04188 | .08426 |
|  | chronic | -.10566^*^ | .031760 | .001 | -.16872 | -.04259 |
| sub_acute | acute | -.02119 | .031760 | .506 | -.08426 | .04188 |
|  | chronic | -.12684^*^ | .031760 | .000 | -.18991 | -.06378 |
| chronic | acute | .10566^*^ | .031760 | .001 | .04259 | .16872 |
|  | sub_acute | .12684^*^ | .031760 | .000 | .06378 | .18991 |
| Based on observed means.  The error term is Mean Square(Error) = .016. | | | | | | |
| *. The mean difference is significant at the 0.05 level. | | | | | | |

SORT CASES BY vaccine_type.

SPLIT FILE LAYERED BY vaccine_type.

UNIANOVA optical_density BY disease_stage

/METHOD=SSTYPE(3)

/INTERCEPT=INCLUDE

/POSTHOC=disease_stage(LSD)

/CRITERIA=ALPHA(0.05)

/DESIGN=disease_stage.

**Univariate Analysis of Variance**

| **Notes** | | |
| --- | --- | --- |
| Output Created | | 30-JUL-2018 11:15:21 |
| Comments | |  |
| Input | Data | C:\Users\DNthiwa\OneDrive - CGIAR\Documents\mydata\Lutta data\lutta_data.sav |
|  | Active Dataset | DataSet2 |
|  | Filter | <none> |
|  | Weight | <none> |
|  | Split File | vaccine_type |
|  | N of Rows in Working Data File | 128 |
| Missing Value Handling | Definition of Missing | User-defined missing values are treated as missing. |
|  | Cases Used | Statistics are based on all cases with valid data for all variables in the model. |
| Syntax | | UNIANOVA optical_density BY disease_stage  /METHOD=SSTYPE(3)  /INTERCEPT=INCLUDE  /POSTHOC=disease_stage(LSD)  /CRITERIA=ALPHA(0.05)  /DESIGN=disease_stage. |
| Resources | Processor Time | 00:00:00.02 |
|  | Elapsed Time | 00:00:00.02 |

| **Between-Subjects Factors** | | | | |
| --- | --- | --- | --- | --- |
| vaccine_type | | | Value Label | N |
| 397 | disease_stage | 1 | acute | 8 |
|  |  | 2 | sub_acute | 8 |
|  |  | 3 | chronic | 8 |
| 636 | disease_stage | 1 | acute | 8 |
|  |  | 2 | sub_acute | 8 |
|  |  | 3 | chronic | 8 |
| 653 | disease_stage | 1 | acute | 8 |
|  |  | 2 | sub_acute | 8 |
|  |  | 3 | chronic | 8 |
| LppB | disease_stage | 1 | acute | 8 |
|  |  | 2 | sub_acute | 8 |
|  |  | 3 | chronic | 8 |

| **Tests of Between-Subjects Effects** | | | | | | |
| --- | --- | --- | --- | --- | --- | --- |
| Dependent Variable: optical_density | | | | | | |
| vaccine_type | Source | Type III Sum of Squares | df | Mean Square | F | Sig. |
| 397 | Corrected Model | .024^a^ | 2 | .012 | .742 | .488 |
|  | Intercept | 1.054 | 1 | 1.054 | 66.329 | .000 |
|  | disease_stage | .024 | 2 | .012 | .742 | .488 |
|  | Error | .334 | 21 | .016 |  |  |
|  | Total | 1.411 | 24 |  |  |  |
|  | Corrected Total | .357 | 23 |  |  |  |
| 636 | Corrected Model | .278^b^ | 2 | .139 | 6.904 | .005 |
|  | Intercept | 2.028 | 1 | 2.028 | 100.744 | .000 |
|  | disease_stage | .278 | 2 | .139 | 6.904 | .005 |
|  | Error | .423 | 21 | .020 |  |  |
|  | Total | 2.728 | 24 |  |  |  |
|  | Corrected Total | .701 | 23 |  |  |  |
| 653 | Corrected Model | .025^c^ | 2 | .012 | 1.758 | .197 |
|  | Intercept | 1.153 | 1 | 1.153 | 163.857 | .000 |
|  | disease_stage | .025 | 2 | .012 | 1.758 | .197 |
|  | Error | .148 | 21 | .007 |  |  |
|  | Total | 1.326 | 24 |  |  |  |
|  | Corrected Total | .173 | 23 |  |  |  |
| LppB | Corrected Model | .064^d^ | 2 | .032 | 1.698 | .207 |
|  | Intercept | 1.666 | 1 | 1.666 | 88.015 | .000 |
|  | disease_stage | .064 | 2 | .032 | 1.698 | .207 |
|  | Error | .397 | 21 | .019 |  |  |
|  | Total | 2.128 | 24 |  |  |  |
|  | Corrected Total | .462 | 23 |  |  |  |
| a. R Squared = .066 (Adjusted R Squared = -.023) | | | | | | |
| b. R Squared = .397 (Adjusted R Squared = .339) | | | | | | |
| c. R Squared = .143 (Adjusted R Squared = .062) | | | | | | |
| d. R Squared = .139 (Adjusted R Squared = .057) | | | | | | |

**Post Hoc Tests**

**disease_stage**

| **Multiple Comparisons** | | | | | | | |
| --- | --- | --- | --- | --- | --- | --- | --- |
| Dependent Variable: optical_density | | | | | | | |
| LSD | | | | | | | |
| vaccine_type | (I) disease_stage | (J) disease_stage | Mean Difference (I-J) | Std. Error | Sig. | 95% Confidence Interval | |
|  |  |  |  |  |  | Lower Bound | Upper Bound |
| 397 | acute | sub_acute | .02713 | .063022 | .671 | -.10394 | .15819 |
|  |  | chronic | -.04863 | .063022 | .449 | -.17969 | .08244 |
|  | sub_acute | acute | -.02713 | .063022 | .671 | -.15819 | .10394 |
|  |  | chronic | -.07575 | .063022 | .243 | -.20681 | .05531 |
|  | chronic | acute | .04863 | .063022 | .449 | -.08244 | .17969 |
|  |  | sub_acute | .07575 | .063022 | .243 | -.05531 | .20681 |
| 636 | acute | sub_acute | .04100 | .070935 | .569 | -.10652 | .18852 |
|  |  | chronic | -.20500^*^ | .070935 | .009 | -.35252 | -.05748 |
|  | sub_acute | acute | -.04100 | .070935 | .569 | -.18852 | .10652 |
|  |  | chronic | -.24600^*^ | .070935 | .002 | -.39352 | -.09848 |
|  | chronic | acute | .20500^*^ | .070935 | .009 | .05748 | .35252 |
|  |  | sub_acute | .24600^*^ | .070935 | .002 | .09848 | .39352 |
| 653 | acute | sub_acute | .00562 | .041947 | .895 | -.08161 | .09286 |
|  |  | chronic | -.06513 | .041947 | .135 | -.15236 | .02211 |
|  | sub_acute | acute | -.00562 | .041947 | .895 | -.09286 | .08161 |
|  |  | chronic | -.07075 | .041947 | .106 | -.15798 | .01648 |
|  | chronic | acute | .06513 | .041947 | .135 | -.02211 | .15236 |
|  |  | sub_acute | .07075 | .041947 | .106 | -.01648 | .15798 |
| LppB | acute | sub_acute | .01100 | .068787 | .874 | -.13205 | .15405 |
|  |  | chronic | -.10388 | .068787 | .146 | -.24693 | .03918 |
|  | sub_acute | acute | -.01100 | .068787 | .874 | -.15405 | .13205 |
|  |  | chronic | -.11487 | .068787 | .110 | -.25793 | .02818 |
|  | chronic | acute | .10388 | .068787 | .146 | -.03918 | .24693 |
|  |  | sub_acute | .11487 | .068787 | .110 | -.02818 | .25793 |
| Based on observed means.  The error term is Mean Square(Error) = .019. | | | | | | | |
| *. The mean difference is significant at the 0.05 level. | | | | | | | |

**Homogeneous Subsets**

SORT CASES BY vaccine_type.

SPLIT FILE LAYERED BY vaccine_type.

SORT CASES BY innoculation.

SPLIT FILE LAYERED BY innoculation.

UNIANOVA optical_density BY disease_stage

/METHOD=SSTYPE(3)

/INTERCEPT=INCLUDE

/POSTHOC=disease_stage(LSD)

/CRITERIA=ALPHA(0.05)

/DESIGN=disease_stage.

**Univariate Analysis of Variance**

| **Notes** | | |
| --- | --- | --- |
| Output Created | | 30-JUL-2018 11:18:41 |
| Comments | |  |
| Input | Data | C:\Users\DNthiwa\OneDrive - CGIAR\Documents\mydata\Lutta data\lutta_data.sav |
|  | Active Dataset | DataSet2 |
|  | Filter | <none> |
|  | Weight | <none> |
|  | Split File | innoculation |
|  | N of Rows in Working Data File | 128 |
| Missing Value Handling | Definition of Missing | User-defined missing values are treated as missing. |
|  | Cases Used | Statistics are based on all cases with valid data for all variables in the model. |
| Syntax | | UNIANOVA optical_density BY disease_stage  /METHOD=SSTYPE(3)  /INTERCEPT=INCLUDE  /POSTHOC=disease_stage(LSD)  /CRITERIA=ALPHA(0.05)  /DESIGN=disease_stage. |
| Resources | Processor Time | 00:00:00.00 |
|  | Elapsed Time | 00:00:00.01 |

| **Warnings** |
| --- |
| No valid cases were found in split file innoculation = pre-inoculation |

| **Between-Subjects Factors** | | | | |
| --- | --- | --- | --- | --- |
| innoculation | | | Value Label | N |
| post-inoculation | disease_stage | 1 | acute | 32 |
|  |  | 2 | sub_acute | 32 |
|  |  | 3 | chronic | 32 |

| **Tests of Between-Subjects Effects** | | | | | | |
| --- | --- | --- | --- | --- | --- | --- |
| Dependent Variable: optical_density | | | | | | |
| innoculation | Source | Type III Sum of Squares | df | Mean Square | F | Sig. |
| post-inoculation | Corrected Model | .295^a^ | 2 | .148 | 9.154 | .000 |
|  | Intercept | 5.796 | 1 | 5.796 | 359.147 | .000 |
|  | disease_stage | .295 | 2 | .148 | 9.154 | .000 |
|  | Error | 1.501 | 93 | .016 |  |  |
|  | Total | 7.593 | 96 |  |  |  |
|  | Corrected Total | 1.796 | 95 |  |  |  |
| a. R Squared = .164 (Adjusted R Squared = .147) | | | | | | |

**Post Hoc Tests**

**disease_stage**

| **Multiple Comparisons** | | | | | | | |
| --- | --- | --- | --- | --- | --- | --- | --- |
| Dependent Variable: optical_density | | | | | | | |
| LSD | | | | | | | |
| innoculation | (I) disease_stage | (J) disease_stage | Mean Difference (I-J) | Std. Error | Sig. | 95% Confidence Interval | |
|  |  |  |  |  |  | Lower Bound | Upper Bound |
| post-inoculation | acute | sub_acute | .02119 | .031760 | .506 | -.04188 | .08426 |
|  |  | chronic | -.10566^*^ | .031760 | .001 | -.16872 | -.04259 |
|  | sub_acute | acute | -.02119 | .031760 | .506 | -.08426 | .04188 |
|  |  | chronic | -.12684^*^ | .031760 | .000 | -.18991 | -.06378 |
|  | chronic | acute | .10566^*^ | .031760 | .001 | .04259 | .16872 |
|  |  | sub_acute | .12684^*^ | .031760 | .000 | .06378 | .18991 |
| Based on observed means.  The error term is Mean Square(Error) = .016. | | | | | | | |
| *. The mean difference is significant at the 0.05 level. | | | | | | | |

**Homogeneous Subsets**

SPLIT FILE OFF.

UNIANOVA optical_density BY innoculation

/METHOD=SSTYPE(3)

/INTERCEPT=INCLUDE

/CRITERIA=ALPHA(0.05)

/DESIGN=innoculation.

**Univariate Analysis of Variance**

| **Notes** | | |
| --- | --- | --- |
| Output Created | | 30-JUL-2018 11:20:34 |
| Comments | |  |
| Input | Data | C:\Users\DNthiwa\OneDrive - CGIAR\Documents\mydata\Lutta data\lutta_data.sav |
|  | Active Dataset | DataSet2 |
|  | Filter | <none> |
|  | Weight | <none> |
|  | Split File | <none> |
|  | N of Rows in Working Data File | 128 |
| Missing Value Handling | Definition of Missing | User-defined missing values are treated as missing. |
|  | Cases Used | Statistics are based on all cases with valid data for all variables in the model. |
| Syntax | | UNIANOVA optical_density BY innoculation  /METHOD=SSTYPE(3)  /INTERCEPT=INCLUDE  /CRITERIA=ALPHA(0.05)  /DESIGN=innoculation. |
| Resources | Processor Time | 00:00:00.00 |
|  | Elapsed Time | 00:00:00.00 |

| **Between-Subjects Factors** | | | |
| --- | --- | --- | --- |
|  | | Value Label | N |
| innoculation | 1 | pre-inoculation | 32 |
|  | 2 | post-inoculation | 96 |

| **Tests of Between-Subjects Effects** | | | | | |
| --- | --- | --- | --- | --- | --- |
| Dependent Variable: optical_density | | | | | |
| Source | Type III Sum of Squares | df | Mean Square | F | Sig. |
| Corrected Model | .474^a^ | 1 | .474 | 32.506 | .000 |
| Intercept | 2.956 | 1 | 2.956 | 202.929 | .000 |
| innoculation | .474 | 1 | .474 | 32.506 | .000 |
| Error | 1.836 | 126 | .015 |  |  |
| Total | 7.986 | 128 |  |  |  |
| Corrected Total | 2.309 | 127 |  |  |  |
| a. R Squared = .205 (Adjusted R Squared = .199) | | | | | |

SORT CASES BY innoculation.

SPLIT FILE LAYERED BY innoculation.

UNIANOVA optical_density BY vaccine_type

/METHOD=SSTYPE(3)

/INTERCEPT=INCLUDE

/POSTHOC=vaccine_type(LSD)

/CRITERIA=ALPHA(0.05)

/DESIGN=vaccine_type.

**Univariate Analysis of Variance**

| **Notes** | | |
| --- | --- | --- |
| Output Created | | 30-JUL-2018 11:24:46 |
| Comments | |  |
| Input | Data | C:\Users\DNthiwa\OneDrive - CGIAR\Documents\mydata\Lutta data\lutta_data.sav |
|  | Active Dataset | DataSet2 |
|  | Filter | <none> |
|  | Weight | <none> |
|  | Split File | innoculation |
|  | N of Rows in Working Data File | 128 |
| Missing Value Handling | Definition of Missing | User-defined missing values are treated as missing. |
|  | Cases Used | Statistics are based on all cases with valid data for all variables in the model. |
| Syntax | | UNIANOVA optical_density BY vaccine_type  /METHOD=SSTYPE(3)  /INTERCEPT=INCLUDE  /POSTHOC=vaccine_type(LSD)  /CRITERIA=ALPHA(0.05)  /DESIGN=vaccine_type. |
| Resources | Processor Time | 00:00:00.02 |
|  | Elapsed Time | 00:00:00.02 |

| **Between-Subjects Factors** | | | | |
| --- | --- | --- | --- | --- |
| innoculation | | | Value Label | N |
| pre-inoculation | vaccine_type | 1 | 397 | 8 |
|  |  | 2 | 636 | 8 |
|  |  | 3 | 653 | 8 |
|  |  | 4 | LppB | 8 |
| post-inoculation | vaccine_type | 1 | 397 | 24 |
|  |  | 2 | 636 | 24 |
|  |  | 3 | 653 | 24 |
|  |  | 4 | LppB | 24 |

| **Tests of Between-Subjects Effects** | | | | | | |
| --- | --- | --- | --- | --- | --- | --- |
| Dependent Variable: optical_density | | | | | | |
| innoculation | Source | Type III Sum of Squares | df | Mean Square | F | Sig. |
| pre-inoculation | Corrected Model | .001^a^ | 3 | .000 | .159 | .923 |
|  | Intercept | .354 | 1 | .354 | 257.643 | .000 |
|  | vaccine_type | .001 | 3 | .000 | .159 | .923 |
|  | Error | .039 | 28 | .001 |  |  |
|  | Total | .394 | 32 |  |  |  |
|  | Corrected Total | .039 | 31 |  |  |  |
| post-inoculation | Corrected Model | .104^b^ | 3 | .035 | 1.891 | .137 |
|  | Intercept | 5.796 | 1 | 5.796 | 315.146 | .000 |
|  | vaccine_type | .104 | 3 | .035 | 1.891 | .137 |
|  | Error | 1.692 | 92 | .018 |  |  |
|  | Total | 7.593 | 96 |  |  |  |
|  | Corrected Total | 1.796 | 95 |  |  |  |
| a. R Squared = .017 (Adjusted R Squared = -.089) | | | | | | |
| b. R Squared = .058 (Adjusted R Squared = .027) | | | | | | |

**Post Hoc Tests**

**vaccine_type**

| **Multiple Comparisons** | | | | | | | |
| --- | --- | --- | --- | --- | --- | --- | --- |
| Dependent Variable: optical_density | | | | | | | |
| LSD | | | | | | | |
| innoculation | (I) vaccine_type | (J) vaccine_type | Mean Difference (I-J) | Std. Error | Sig. | 95% Confidence Interval | |
|  |  |  |  |  |  | Lower Bound | Upper Bound |
| pre-inoculation | 397 | 636 | .01137 | .018546 | .545 | -.02662 | .04937 |
|  |  | 653 | .00875 | .018546 | .641 | -.02924 | .04674 |
|  |  | LppB | .00288 | .018546 | .878 | -.03512 | .04087 |
|  | 636 | 397 | -.01137 | .018546 | .545 | -.04937 | .02662 |
|  |  | 653 | -.00262 | .018546 | .888 | -.04062 | .03537 |
|  |  | LppB | -.00850 | .018546 | .650 | -.04649 | .02949 |
|  | 653 | 397 | -.00875 | .018546 | .641 | -.04674 | .02924 |
|  |  | 636 | .00262 | .018546 | .888 | -.03537 | .04062 |
|  |  | LppB | -.00588 | .018546 | .754 | -.04387 | .03212 |
|  | LppB | 397 | -.00288 | .018546 | .878 | -.04087 | .03512 |
|  |  | 636 | .00850 | .018546 | .650 | -.02949 | .04649 |
|  |  | 653 | .00588 | .018546 | .754 | -.03212 | .04387 |
| post-inoculation | 397 | 636 | -.08112^*^ | .039150 | .041 | -.15888 | -.00337 |
|  |  | 653 | -.00967 | .039150 | .806 | -.08742 | .06809 |
|  |  | LppB | -.05392 | .039150 | .172 | -.13167 | .02384 |
|  | 636 | 397 | .08112^*^ | .039150 | .041 | .00337 | .15888 |
|  |  | 653 | .07146 | .039150 | .071 | -.00630 | .14921 |
|  |  | LppB | .02721 | .039150 | .489 | -.05055 | .10496 |
|  | 653 | 397 | .00967 | .039150 | .806 | -.06809 | .08742 |
|  |  | 636 | -.07146 | .039150 | .071 | -.14921 | .00630 |
|  |  | LppB | -.04425 | .039150 | .261 | -.12200 | .03350 |
|  | LppB | 397 | .05392 | .039150 | .172 | -.02384 | .13167 |
|  |  | 636 | -.02721 | .039150 | .489 | -.10496 | .05055 |
|  |  | 653 | .04425 | .039150 | .261 | -.03350 | .12200 |
| Based on observed means.  The error term is Mean Square(Error) = .018. | | | | | | | |
| *. The mean difference is significant at the 0.05 level. | | | | | | | |

**Homogeneous Subsets**

UNIANOVA optical_density BY innoculation

/METHOD=SSTYPE(3)

/INTERCEPT=INCLUDE

/CRITERIA=ALPHA(0.05)

/DESIGN=innoculation.

**Univariate Analysis of Variance**

| **Notes** | | |
| --- | --- | --- |
| Output Created | | 30-JUL-2018 12:05:29 |
| Comments | |  |
| Input | Data | C:\Users\DNthiwa\OneDrive - CGIAR\Documents\mydata\Lutta data\lutta_data.sav |
|  | Active Dataset | DataSet1 |
|  | Filter | <none> |
|  | Weight | <none> |
|  | Split File | <none> |
|  | N of Rows in Working Data File | 128 |
| Missing Value Handling | Definition of Missing | User-defined missing values are treated as missing. |
|  | Cases Used | Statistics are based on all cases with valid data for all variables in the model. |
| Syntax | | UNIANOVA optical_density BY innoculation  /METHOD=SSTYPE(3)  /INTERCEPT=INCLUDE  /CRITERIA=ALPHA(0.05)  /DESIGN=innoculation. |
| Resources | Processor Time | 00:00:00.02 |
|  | Elapsed Time | 00:00:00.02 |

[DataSet1] C:\Users\DNthiwa\OneDrive - CGIAR\Documents\mydata\Lutta data\lutta_data.sav

| **Between-Subjects Factors** | | | |
| --- | --- | --- | --- |
|  | | Value Label | N |
| innoculation | 1 | pre-inoculation | 32 |
|  | 2 | post-inoculation | 96 |

| **Tests of Between-Subjects Effects** | | | | | |
| --- | --- | --- | --- | --- | --- |
| Dependent Variable: optical_density | | | | | |
| Source | Type III Sum of Squares | df | Mean Square | F | Sig. |
| Corrected Model | .474^a^ | 1 | .474 | 32.506 | .000 |
| Intercept | 2.956 | 1 | 2.956 | 202.929 | .000 |
| innoculation | .474 | 1 | .474 | 32.506 | .000 |
| Error | 1.836 | 126 | .015 |  |  |
| Total | 7.986 | 128 |  |  |  |
| Corrected Total | 2.309 | 127 |  |  |  |
| a. R Squared = .205 (Adjusted R Squared = .199) | | | | | |

UNIANOVA optical_density BY innoculation

/METHOD=SSTYPE(3)

/INTERCEPT=INCLUDE

/POSTHOC=innoculation(LSD)

/CRITERIA=ALPHA(0.05)

/DESIGN=innoculation.

**Univariate Analysis of Variance**

| **Notes** | | |
| --- | --- | --- |
| Output Created | | 30-JUL-2018 12:05:45 |
| Comments | |  |
| Input | Data | C:\Users\DNthiwa\OneDrive - CGIAR\Documents\mydata\Lutta data\lutta_data.sav |
|  | Active Dataset | DataSet1 |
|  | Filter | <none> |
|  | Weight | <none> |
|  | Split File | <none> |
|  | N of Rows in Working Data File | 128 |
| Missing Value Handling | Definition of Missing | User-defined missing values are treated as missing. |
|  | Cases Used | Statistics are based on all cases with valid data for all variables in the model. |
| Syntax | | UNIANOVA optical_density BY innoculation  /METHOD=SSTYPE(3)  /INTERCEPT=INCLUDE  /POSTHOC=innoculation(LSD)  /CRITERIA=ALPHA(0.05)  /DESIGN=innoculation. |
| Resources | Processor Time | 00:00:00.00 |
|  | Elapsed Time | 00:00:00.04 |

| **Warnings** |
| --- |
| Post hoc tests are not performed for innoculation because there are fewer than three groups. |

| **Between-Subjects Factors** | | | |
| --- | --- | --- | --- |
|  | | Value Label | N |
| innoculation | 1 | pre-inoculation | 32 |
|  | 2 | post-inoculation | 96 |

| **Tests of Between-Subjects Effects** | | | | | |
| --- | --- | --- | --- | --- | --- |
| Dependent Variable: optical_density | | | | | |
| Source | Type III Sum of Squares | df | Mean Square | F | Sig. |
| Corrected Model | .474^a^ | 1 | .474 | 32.506 | .000 |
| Intercept | 2.956 | 1 | 2.956 | 202.929 | .000 |
| innoculation | .474 | 1 | .474 | 32.506 | .000 |
| Error | 1.836 | 126 | .015 |  |  |
| Total | 7.986 | 128 |  |  |  |
| Corrected Total | 2.309 | 127 |  |  |  |
| a. R Squared = .205 (Adjusted R Squared = .199) | | | | | |

SORT CASES BY innoculation.

SPLIT FILE LAYERED BY innoculation.

UNIANOVA optical_density BY innoculation

/METHOD=SSTYPE(3)

/INTERCEPT=INCLUDE

/POSTHOC=innoculation(LSD)

/CRITERIA=ALPHA(0.05)

/DESIGN=innoculation.

**Univariate Analysis of Variance**

| **Notes** | | |
| --- | --- | --- |
| Output Created | | 30-JUL-2018 12:06:19 |
| Comments | |  |
| Input | Data | C:\Users\DNthiwa\OneDrive - CGIAR\Documents\mydata\Lutta data\lutta_data.sav |
|  | Active Dataset | DataSet1 |
|  | Filter | <none> |
|  | Weight | <none> |
|  | Split File | innoculation |
|  | N of Rows in Working Data File | 128 |
| Missing Value Handling | Definition of Missing | User-defined missing values are treated as missing. |
|  | Cases Used | Statistics are based on all cases with valid data for all variables in the model. |
| Syntax | | UNIANOVA optical_density BY innoculation  /METHOD=SSTYPE(3)  /INTERCEPT=INCLUDE  /POSTHOC=innoculation(LSD)  /CRITERIA=ALPHA(0.05)  /DESIGN=innoculation. |
| Resources | Processor Time | 00:00:00.02 |
|  | Elapsed Time | 00:00:00.02 |

| **Warnings** |
| --- |
| Post hoc tests are not performed for innoculation in split file innoculation=pre-inoculation because there are fewer than three groups. |
| Post hoc tests are not performed for innoculation in split file innoculation=post-inoculation because there are fewer than three groups. |

| **Between-Subjects Factors** | | | | |
| --- | --- | --- | --- | --- |
| innoculation | | | Value Label | N |
| pre-inoculation | innoculation | 1 | pre-inoculation | 32 |
| post-inoculation | innoculation | 2 | post-inoculation | 96 |

| **Tests of Between-Subjects Effects** | | | | | | |
| --- | --- | --- | --- | --- | --- | --- |
| Dependent Variable: optical_density | | | | | | |
| innoculation | Source | Type III Sum of Squares | df | Mean Square | F | Sig. |
| pre-inoculation | Corrected Model | .000^a^ | 0 | . | . | . |
|  | Intercept | .354 | 1 | .354 | 280.473 | .000 |
|  | innoculation | .000 | 0 | . | . | . |
|  | Error | .039 | 31 | .001 |  |  |
|  | Total | .394 | 32 |  |  |  |
|  | Corrected Total | .039 | 31 |  |  |  |
| post-inoculation | Corrected Model | .000^a^ | 0 | . | . | . |
|  | Intercept | 5.796 | 1 | 5.796 | 306.525 | .000 |
|  | innoculation | .000 | 0 | . | . | . |
|  | Error | 1.796 | 95 | .019 |  |  |
|  | Total | 7.593 | 96 |  |  |  |
|  | Corrected Total | 1.796 | 95 |  |  |  |
| a. R Squared = .000 (Adjusted R Squared = .000) | | | | | | |
